# Exogenous Jaagsiekte sheep retrovirus (JSRV) Inner Mongolia strain: whole-genome characterization and viral particle packaging

**DOI:** 10.3389/fvets.2025.1608822

**Published:** 2025-08-18

**Authors:** Pei Zhang, Yu Wang, Xujie Duan, Sixu Chen, Xiaoyue Du, Anyu Bao, XinQi Ma, Yufei Zhang, Shuying Liu

**Affiliations:** ^1^College of Veterinary Medicine, Inner Mongolia Agricultural University, Hohhot, China; ^2^Key Laboratory of Veterinary Fundamentals and Disease Prevention and Control for Herbivorous Livestock, Hohhot, China

**Keywords:** ovine pulmonary adenocarcinoma, exogenous Jaagsiekte sheep retrovirus, endogenous Jaagsiekte sheep retrovirus, genomic sequencing, phylogenetic analysis, virus particles

## Abstract

**Introduction:**

Ovine pulmonary adenocarcinoma (OPA) is a contagious lung tumor caused by the exogenous Jaagsiekte sheep retrovirus (exJSRV). Analysing the genome of the pathogen is crucial for developing OPA prevention and control measures. Due to the absence of exogenous genomic JSRV-related information in Inner Mongolia, we aimed to establish a specific technique for exJSRV genomic amplification.

**Methods:**

Target virions were purified using U3 hn-PCR (hemi-nested PCR) combined with density gradient centrifugation. Specific reverse transcription primers were designed using the low-identity region of the internal and external genome, combined with long fragment PCR and 3′RACE technology, and the full-length genome of exogenous JSRV from Inner Mongolia was successfully obtained.

**Results:**

Exogenous molecular characteristics were found in the long terminal repeat(LTR)-U3 region, gag-variable region 1/2(VR1/VR2) and env-VR3, and was 98.8% identical to the Chinese JSRV-C1, which was significantly higher than that of foreign isolates (93.05–95.84%) and enogenous Jaagsiekte sheep retrovirus (enJSRV) (88.73–92.26%). Phylogenetic analysis showed that NMJS12 and exogenous JSRV-C1 were located in the same evolutionary clade. Accordingly, the whole genome eukaryotic expression plasmid was successfully constructed and viral particle packaging was achieved in 293T cells.

**Conclusion:**

Altogether, this study represents the first elucidation of the complete genome of exogenous JSRV in Inner Mongolia, China and provides a critical material foundation for antiviral target screening and research on OPA pathogenesis.

## 1 Introduction

Ovine pulmonary adenocarcinoma (OPA) is a chronic, progressive, infectious, and naturally occurring neoplastic disease caused by infection with the exogenous Jaagsiekte sheep retrovirus (exJSRV). The pathologic features of exJSRV are mainly neoplastic hyperplasia of alveolar type II epithelial and bronchial Clara cells (1). Since the it was first identified in South Africa in 1825, OPA has become globally distributed, with widespread prevalence in sheep populations across Europe, Africa, the Americas, and Asia (2, 3). Notably, in high-incidence regions, such as South Africa and the United Kingdom, flock infection rates can reach 70% (2, 3). Since the first case of OPA was confirmed in Lanzhou, Gansu Province, China, in 1951 (4, 5), exJSRV has spread to major sheep-raising areas, such as Inner Mongolia, Qinghai, and Xinjiang (6–9). The persistent dissemination of OPA indicates that interregional livestock trade plays a pivotal role in its transmission. Owing to its severe impact on animal health and the livestock economy, the World Organization for Animal Health (WOAH) has classified OPA as a significant infectious disease (10).

As a major meat sheep production base in China, the Inner Mongolia Autonomous Region faces an elevated risk of exJSRV transmission due to its intensive farming practices (11). Due to the lack of effective therapeutic interventions against exJSRV infection, infected flocks often suffer high mortality rates caused by progressive immunosuppression and secondary infections, severely threatening the sustainable development of the local sheep industry. Therefore, elucidating the phylogenetic relationship of exJSRV strains prevalent in Inner Mongolia is critical for formulating targeted prevention and control strategies. Notably, the clinical manifestations of OPA (e.g., coughing, dyspnea, and serous nasal discharge) and its histopathological features are markedly similar to those of human bronchioloalveolar carcinoma (BAC) (3, 12). This striking resemblance establishes OPA as a vital animal model for studying lung cancer pathogenesis and exploring potential therapeutic targets.

ExJSRV belongs to the genus *Betaretrovirus* within the family *Retroviridae*. The genome of this virus includes a 7.5 kb single-stranded positive-sense RNA and encodes canonical retroviral essential genes (*gag-pro-pol-env*) and long terminal repeats (LTRs) required for viral replication (13). The Env protein of exJSRV acts as its oncogene. The surface subunit (SU) mediates viral entry through interaction with the host receptor hyaluronidase 2 (Hyal2) while the cytoplasmic tail of the transmembrane (TM) glycoprotein drives malignant transformation. The primary mechanism involves the TM subunit recruiting host adaptor proteins, such as RaLBP1, via its “YXXM (where X represents any amino acid)” motif, leading to aberrant activation of the Akt/mTOR and MAPK signaling pathways (14–16). Notably, endogenous JSRVs (enJSRVs), which are integrated into the sheep genome through Mendelian inheritance, lack oncogenic potential due to the absence of the “YXXM (where X represents any amino acid)” motif in their Env proteins (17, 18). The high genomic sequence identity between exJSRV and enJSRV poses significant technical challenges for developing molecular-specific diagnostic methods targeting exJSRV strains.

To date, only two exJSRV full-genome sequences from Inner Mongolia (ON204347) and Xinjiang (KP691837) have been reported in China (19); however, their genetic evolutionary characteristics and pathogenic differences remain uninvestigated systematically. In this study, we confirmed three natural exJSRV infection cases in Inner.

Mongolia by integrating histopathological diagnosis with molecular detection techniques. Moreover, we successfully obtained the full-length viral genome using sucrose gradient centrifugation, long-fragment PCR, and 3′ rapid amplification of cDNA ends (RACE) technology; constructed a full-length molecular clone through homologous recombination; additionally, we systematically characterized its reverse transcriptase activity and virion morphological features. This study not only addresses the gap in genetic evolutionary data of exJSRV strains in China but also provides a theoretical foundation for elucidating the impact of geographic adaptation-associated mutations on pathogenicity and screening effective therapeutic targets.

## 2 Materials and methods

### 2.1 Source of sample and hematoxylin–eosin (H&E) Staining

In 2020, lung tissues from 25 clinically symptomatic 1–2-year-old Dumeng rams with respiratory distress were collected at a ranch in Inner Mongolia, China. Among these tissues, 7 had tumor-like gross lesions, 5 displayed chronic pulmonary inflammation with mild fibrinous exudation, and 13 had no obvious lesions. Tissues were fixed in 10% neutral buffered formalin or snap-frozen at −80°C. Formalin-fixed specimens were processed following standard pathological protocols. Finally, paraffin-embedded tissues were sectioned into 3–5 μm slices and stained with hematoxylin and eosin (H&E).

### 2.2 RNA extraction and molecular diagnosis using U3 hn-PCR

An appropriate amount of lung tissue was retrieved from storage at −80°C. Total RNA was extracted from the lung tissue using a total RNA extraction kit (Axygen, Union City, California, USA) and reverse-transcribed into cDNA using the Hiscript III First-Strand cDNA Synthesis Kit (Vazyme, Nanjing, China), according to the instructions. The cDNA was then used as a template for subsequent polymerase chain reaction analysis (PCR) with the primers U3-I, U3-III, and U3-VI. The primer sequences are shown in Table 1 (20, 21). PCR was first performed with primers U3-I and U3-III, and a secondary nested PCR was performed with primers U3-I and U3-VI (Table 1). cDNA from U3 hn-PCR-positive samples was amplified with primers env-F and U3-III (Table 1) for further characterization (1). All PCR products were sent to Sangon Biotech Co., Ltd. (Shanghai, China) for sanger sequencing verification.

**Table 1:** Primer sequences for amplifying the full-length genome of NMJSRV.

Primer name	Primer sequences (5′–3′)	Length (bp)
AFRT1	TTTATTACAATGCTATAT	For RT-PCR detection
AFRT2	TAATCAGATTTCCTGG	
AFRT3	GTAACATATTTCTATA	
AFRT4	TACTGATGCGACCCGG	
AFRT5	CAAATACGGGCGTAT	
AFRT6	TTGTTGGAAAAGTACT	
U3-I	GCAGAGTATCAGCCATTTTGGTC	176
U3-III	GAATCAGAGGTCCCTAAGGAAAAC	
U3-VI	TTTCGGGTCCTCTGACGCCTATTGG	133
env-F	GAAATGCTGCATATGAAATATAG	286
NMJSRV-F	GCAGAGTATCAGCCATTTTGGTCTGATC	7,359
NMJSRV-R	TATTCACTACACCCACCGGATTCTTACACAATCACCGG	
3′RACE-F	CCAGGAAATCTGATTATATAAGAATC	122
3′RACE-R	GCTGATACTCTGCTTTATTACAATGCTATATTTATAAAG	
pc-NMJS12-KpnI-F	tggctagttaagcttggtaccGCAGAGTATCAGCCATTTTGGTC	7,393
U3u-NMJS12-R	cttacacaatcacCGGATTCTTATATAATCAGATTTCCTG	
U3d-F	gaatccgGTGATTGTGTAAGAATCCGGTGG	118
U3d-U5-R	ggatcagaccaaaatgACTGATACTCTGCTTTATTACAATGCTATAT	
U3-U5-F	cagcCGTTTTGGTCTGATCCTCTCAAC	139
pc-U5-NotI-R	gccctctagactcgagcggccgcTGCCGCGGCCAGCACAAG	

Underline: known sequence; RT-PCR, reverse transcription polymerase chain reaction; Lowercase letters: the homologous sequences between the vector and the fragment.

### 2.3 Virus enrichment and reverse transcriptase (RT) assay

Lung tissue samples that were confirmed to harbor exJSRV based on sequencing were cryogenically pulverized using liquid nitrogen prior to downstream molecular analyses. A 5-fold volume of PBS was then added to create a suspension. Five freeze–thaw cycles were performed to release the virus. The suspension was centrifuged at 8,000 × *g* for 20 min. Following three additional rounds of centrifugation, the supernatant was collected and centrifuged at 16,743 × *g* for 1 h. The supernatant was ultracentrifuged at 82,992 × *g* for 3 h using a SW32ti rotor (Beckman Coulter Optima L-XP100 ultracentrifuge). The pellet was resuspended in TNE buffer and centrifuged at 82,992 × *g* for 3 h. The virus was purified using a 20–50% (wt/wt) sucrose density gradient and ultracentrifugation. Finally, the purified sample was resuspended in PBS for the RT assays and genomic amplification.

RT activity was quantified by measuring the incorporation of labeled nucleotides into the immobilized RNA template. The digoxin-labeled antibody conjugated with peroxidase was bound to the labeled nucleotides. Peroxidase was used to catalyse ABTS cleavage to obtain the colored product. RT activity was determined by measuring absorbance at 405 nm. Absorbance is directly proportional to RT activity.

### 2.4 Integrated long-range PCR and 3′ RACE strategies for high-resolution sequencing of the exJSRV genome

RNA was extracted from the purified viral samples. Specific reverse transcription primers targeting divergent regions of exJSRV and enJSRV genomes were designed based on sequence variations within their whole-genome alignments. The RNA was subsequently reverse-transcribed into cDNA using the primers described above (Table 1). Long-range PCR was performed using this cDNA, primers NMJSRV-F and NMJSRV-R (Table 1) (1), and the Q5® High-Fidelity DNA Polymerase kit (New England Biolabs, NEB). The PCR products were sequenced for sanger sequencing verification, and the consensus sequences were assembled from the sequencing data. Based on the assembled sequences, specific primers for 3′ RACE were designed (Table 1) to amplify and sequence the 3′ termini.

### 2.5 Sequence and phylogenetic analysis

The amplified fragments were sequenced using overlapping primers NMJSRV-F/R and env-F/u3-III listed in Table 1 via Sanger sequencing. Sequences exhibiting sharp and symmetric peaks without tailing or splitting, presenting single-base signals were prioritized during screening. Secondary criteria required stable baselines without significant fluctuations or high background noise. Finally, complete concordance between forward and reverse sequencing results within overlapping regions was confirmed. Sequences meeting these criteria were aligned with NCBI reference sequences (as shown for exJSRV in Table 2), and target fragments were assembled using the Seqman software in DNASTAR v7.1 software (DNASTAR Inc., Madison, WI, USA) based on positions corresponding to the reference sequences to generate consensus sequences, thereby ensuring data accuracy.

**Table 2:** List of exJSRV and enJSRV used in the complete genome analyses.

exJSRV				enJSRV			
Accession number	Strain name	Year	Country	Accession number	Strain name	Year	Country
M80216	JSRV-SA	1992	USA	AF136224	enJSRV 5f16	2000	South Africa
AF105220	JSRV21	1999	USA	AF153615	enJSRV 5.6A1	2000	South Africa
AF357971	JSRV-JS7	2001	USA	DQ838493	enJSRV-NM	2008	China
KP691837	JSRV-C1	2013	China	EF680311	enJSRV-1	2007	USA
MN161849	DL37	2017	India	EF680310	enJSRV-2	2007	USA
OR729406	SVUJY	2019	India	EF680309	enSRV-3	2007	USA
MW883893	Tonk-1	2020	India	EF680305	enSRV-5	2007	USA
PP707037	FR1296	2008	France	EF680298	enJSRV-7	2007	USA
PP707038	FR1466	2009	France	EF680306	enJSRV-8	2007	USA
PP707039	FR1000	2004	France	EF680307	enJSRV-10	2007	USA
PP707040	FR985	2003	France	EF680303	enJSRV-11	2007	USA
PP707041	FR1037	2006	France	EF680296	enJSRV-13	2007	USA
PP707042	FR1468	2009	France	EF68030	enJSRV-14	2007	USA
PP707043	FR987	2003	France	EF680299	enJSRV-15	2007	USA
PP707044	FR1167	2008	France	EF680300	enJSRV-16	2007	USA
PP707045	FR1762	2012	France	EF680301	enlSRV-18	2007	USA
PP707046	FR1298	2008	France	EF680304	enJSRV-19	2007	USA
PP707047	FR989	2003	France	EF680302	enJSRV-20	2007	USA
PP707048	FR2780	2019	France	EF680312	enJSRV-21	2007	USA
PP707049	FR2586	2016	France	EF680313	enJSRV-23	2007	USA
PP707050	FR2054	2012	France	EF680314	enJSRV-25	2007	USA
PP707051	FR2332	2012	France	EF680297	enJSRV-26	2007	USA
PP707052	FR2529	2014	France	MF175067	HamJ1	2017	United Kingdom
PP707053	FR2334	2012	France	MF175068	HamJ2	2017	United Kingdom
PP707054	FR2334	2012	France	MF175069	HamM	2017	United Kingdom
PP707055	FR1751	2011	France	MF175070	KarJ	2017	United Kingdom
PP646154	FR1481	2012	France	MF175071	KarM	2017	United Kingdom
PP646155	FR2369	2011	France	–	–	–	–

Multiple sequence alignment of nucleotide sequences was performed using the Sequence Demarcation Tool Version 1.3 (SDTv1.3). Nucleotide and amino acid sequence alignments were conducted using the MegAlign software within the DNASTAR v7.1 software. To analyze the genetic evolutionary relationship of NMJS12, phylogenetic analysis was performed via the neighbor-joining method with 1,000 bootstrap replicates in MEGA v11.0 software,^1^ with EF680307.1 (enJSRV10) designated as the outgroup for tree rooting. In addition to this strain, sequences from 55 previously reported retrovirus reference strains were included for comparison (Table 2).

### 2.6 Molecular cloning and viral particle packaging

After obtaining the full-length genome sequence from section 2.6, it was aligned with other exJSRV isolate sequences from NCBI to locate the U3, U5, and R regions. Using the amplicons of NMJSRV-F/NMJSRV-R and 3′RACE-F/3′RACE-R as templates, PCR amplification was performed with the Q5® High-Fidelity DNA Polymerase Kit (New England Biolabs, NEB) and the primer pairs pc-NMJS12-*KpnI*-F/U3u-NMJS12 R, U3d-F/U3d-U5-R, and U3-U5-F/pc-U5-*NotI*-R to generate the corresponding target fragments. Following amplification, the three fragments were cloned into the pcDNA4.0 vector (digested with *KpnI* and *NotI* and containing the CMV strong promoter) via *fusion PCR* and T4 DNA ligase (New England Biolabs, NEB). The primers used are listed in Table 1. The successfully ligated full-length molecular clones were identified by Sanger sequencing using pc-NMJS12-*KpnI* F/U3u-NMJS12-R, U3d-F/U3d-U5-R, and U3-U5-F/pc-U5-*NotI*-R.

The JSRV molecular clone was transfected into 293T cells cultured in medium containing 10% fetal bovine serum (FBS; Gibco, Thermo Fisher Scientific Inc.) using Lip3000 transfection reagent (Thermo Fisher Scientific Inc.). Six hours post transfection, the medium was replaced with fresh complete medium. Culture supernatants were harvested at 24, 48, and 72 h post-transfection. The collected supernatants were then subjected to preliminary clarification via centrifugation (16,743 × *g*, 10 min, 4°C), filtration through 0.45 μm polyethersulfone (PES) membrane filters, and viral concentration via ultracentrifugation at 82,992 × *g* for 3 h using a SW32ti rotor (Beckman Coulter Optima L-XP100 ultracentrifuge). The purified viral particles were aliquoted and cryopreserved at −80°C for downstream applications.

### 2.7 RT assay and electron microscopy

The RT activity was quantified by measuring the incorporation of digoxigenin and biotin-labeled nucleotides in the fixed RNA template provided by the manufacturer (Roche colorimetric Reverse Transcriptase Assay, Merck, Gillingham, UK). The assay involves the binding of digoxigenin-labeled antibody (anti-DIG-POD) conjugated with peroxidase to the labeled nucleotides. Of note, peroxidase catalyzes the cleavage of the substrate, ABTS, to generate a colored reaction product. The RT activity of the sample was determined by measuring the absorbance at 405 nm as absorbance is directly proportional to the RT activity of the sample. At 48 h after transient transfection, 293T cells were fixed with 2.5% glutaraldehyde in 100 mM phosphate buffer (pH 7.2) and observed using an electron microscope (EM).

## 3 Results

### 3.1 Gross pathology

**Figure 1:**
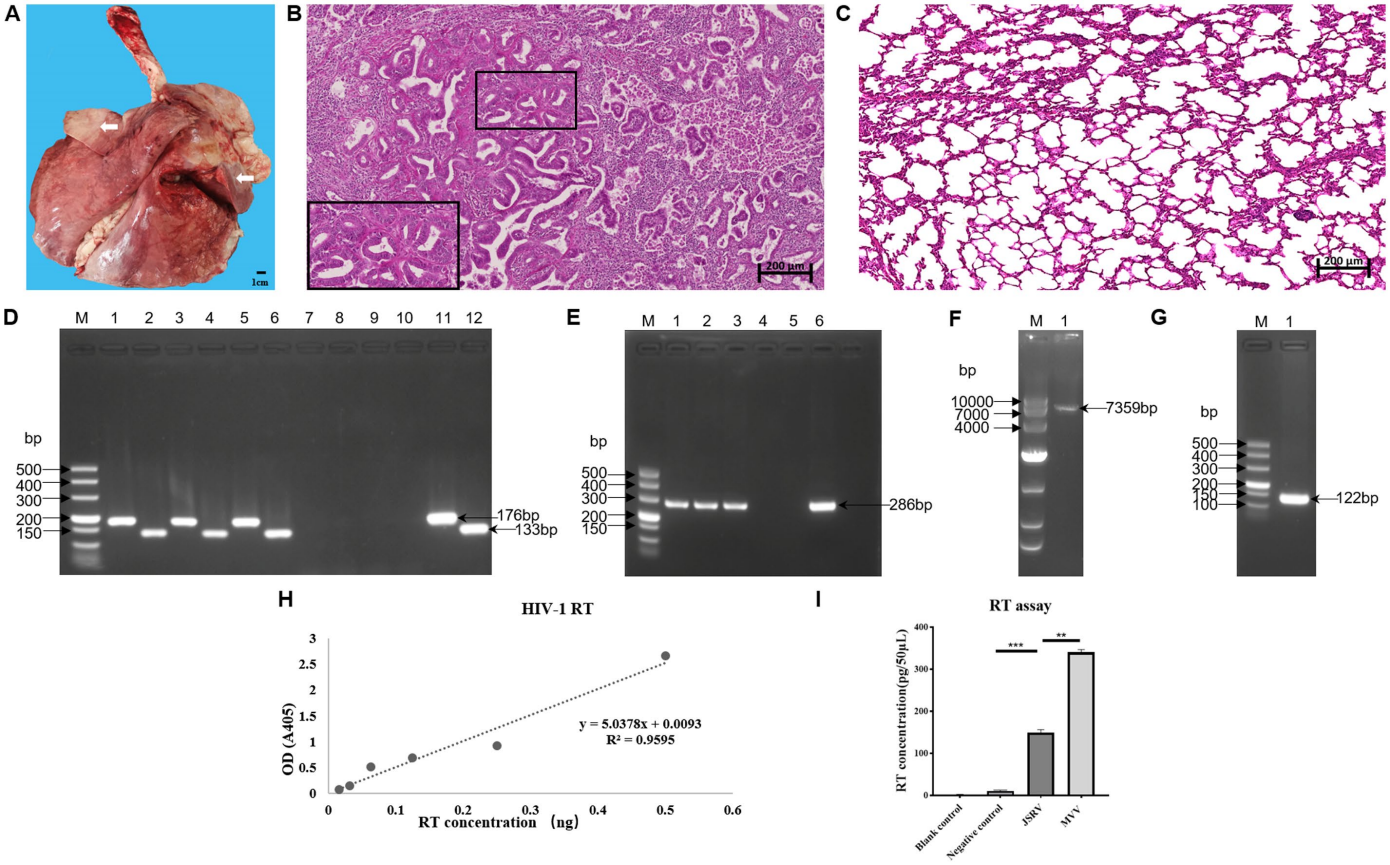
Histological, molecular, and RT assays of exJSRV in sheep lung. **(A)** Gross lung pathologies in JSRV-infected sheep (white arrow). **(B)** Neoplastic proliferative foci (demarcated by black boxes) originating from hyperplasia of alveolar type II epithelial cells (black boxes) based on histopathological analysis. Scale bar: 200 μm. **(C)** In healthy lung tissue, the alveolar architecture is characterized by thin, delicate septa lined by a continuous layer of epithelial cells. Scale bar: 200 μm. **(D)** PCR detection of exJSRV U3; lanes 1–12: lung sample (1–6), blank control (7–8), negative control (9–10), and positive control (11–12). **(E)** PCR detection of exJSRV env; lanes 1–6: lung sample (1–3), blank control (4), negative control (5), and positive control (6). **(F)** PCR amplicon obtained from Long-range PCR. **(G)** Amplicons obtained using the 3′ RACE method. **(H)** Calibration curve of HIV-1 reverse transcriptase. **(I)** Comparative analysis of RT activity levels revealed statistically significant differences (*p < 0.05, **p < 0.01, ***p < 0.001; Student’s *t*-test) between the exJSRV-infected group and the negative control group (uninfected tissues). As anticipated, the positive control group (MVV-infected cell culture supernatants) exhibited robust RT activity, consistent with the lentiviral replication profile of MVV. No detectable RT activity was observed in the blank control group (reagent-only samples), confirming the absence of nonspecific enzymatic interference.

Among the 25 pulmonary tissue samples, 7 were initially screened as positive for suspected OPA characteristics. Subsequent histopathological evaluation combined with U3 hn-PCR led to the diagnosis of 3 cases of exJSRV infection. The infected lungs exhibited marked pulmonary enlargement with localized consolidation during thoracotomy dissection. Furthermore, the cardiac lobes of both lungs had a firm texture with well-demarcated red-grayish white lesions (Figure 1A). Histopathological examination revealed significant hyperplasia of type II alveolar epithelial cells, displaying cuboidal to columnar morphological features protruding into alveolar and bronchiolar lumina (Figures 1B,C). These pathological manifestations align with OPA-specific diagnostic criteria, demonstrating high concordance with its principal histopathological indicators.

### 3.2 PCR, RT assay, and full-length genome sequencing

Three histopathologically confirmed OPA lung tissue samples were analyzed using specific primers targeting both non-coding and coding regions of the pathogen. U3 hn-PCR revealed the expected 176 base pair (bp) and 133 base pair (bp) amplification products (Figure 1D). The env-F/U3-III primer pair also led to successful generation of a 286 bp target band (Figure 1E). No amplification bands were observed in the negative control or blank control samples. Sequencing analysis of the amplified fragments from the three samples revealed 100% sequence identical. Based on these findings, one exJSRV-positive lung tissue sample was selected for subsequent viral particle purification using sucrose density gradient centrifugation.

Based on sucrose density gradient centrifugation analysis, RT activity calculated from the standard curve (Figure 1H) was exclusively detected in the 30–50% (w/w) sucrose gradient fractions (Figure 1I), corresponding to the previously reported buoyant density characteristics of JSRV (1.16–1.18 g/mL) (1, 22). It is noteworthy that the RT concentration was significantly lower than that in the supernatant of cells infected with MVV. The viral particle-containing 30–50% sucrose fractions were subsequently subjected to RNA extraction and targeted reverse transcription into specific cDNA. A 7,359 bp full-length exJSRV genome product was successfully amplified through long-range PCR (Figure 1F). Following sequencing of this product, 3′ RACE primers were designed to generate the expected 122 bp amplification fragment (Figure 1G).

### 3.3 Characteristics of the NMJS12 genome

**Figure 2:**
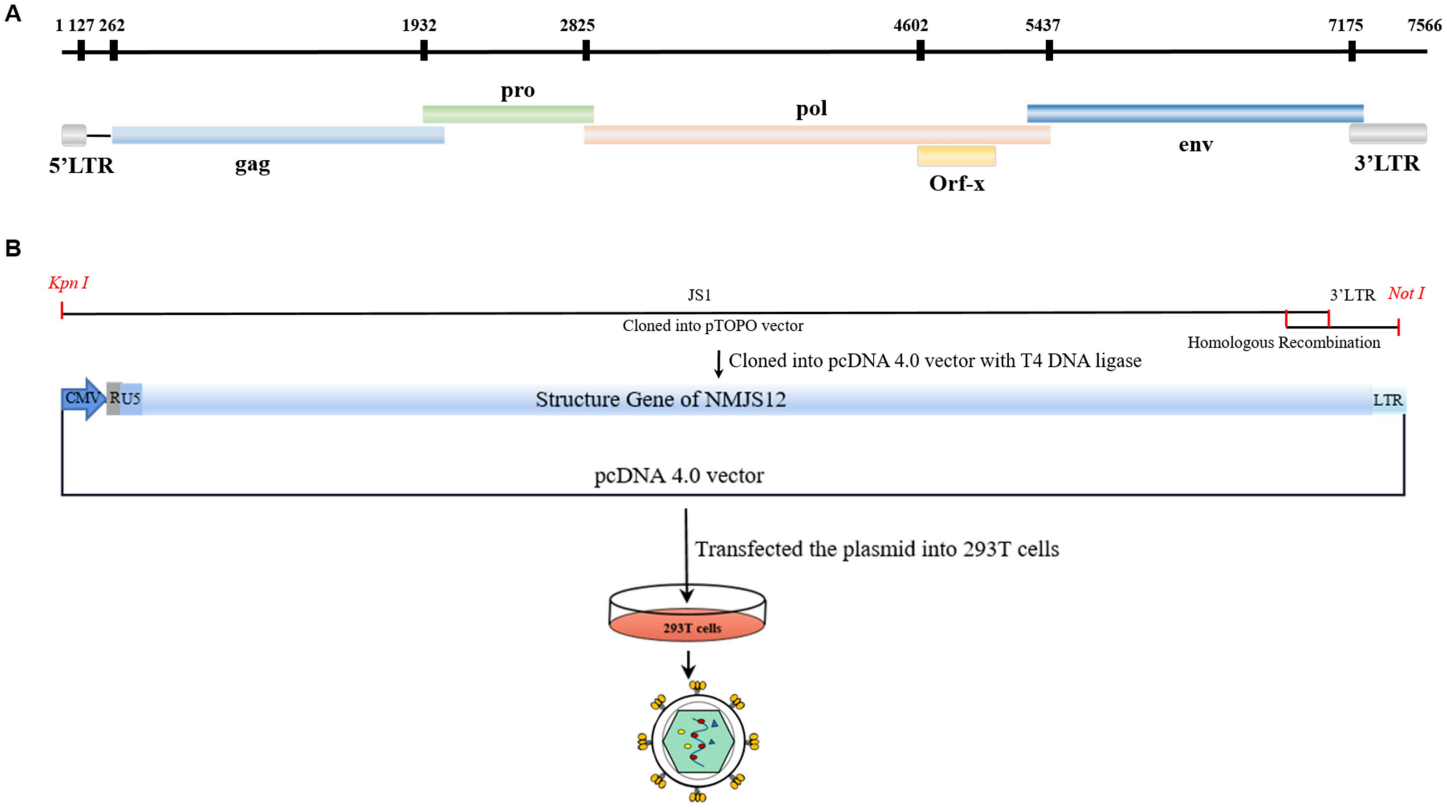
Schematic representation of gene distribution in the NMJSRV genome. **(A)** Removal of the U3 region from the 5′ LTR region of the NMJS12 proviral DNA genome. **(B)** Strategy used to construct the infectious molecular clone of NMJS12. LTR, long terminal repeat.

Sequence assembly using Seqman software produced the complete genomic sequence of the exJSRV NM strain, designated as NMJS12. The nucleotide sequence of the complete genome was deposited in GenBank under accession number ON204347. The NMJS12 provirus was found to comprise 7,833 bp and had a viral genome (R to R) containing 7,454 nucleotide (nt). Moreover, the genome was found to contain five open reading frames (ORFs): the gag gene (position 262 to 2097) encodes 611 amino acids; the pro gene (position 1932 to 2,858) encodes 308 amino acids; the pol gene (position 2,825 to 5,437) encodes 870 amino acids; the orf-x gene (position 4,602 to 5,066) encodes 154 amino acids; and the env gene (position 5,346 to 7,193) encodes 615 amino acids (Table 3, Figure 2A).

**Table 3:** Genome information for NMJS12.

Name	Position	Fragment length (bp)	Amino acid length
5′LTR	1 ~ 127	127	N/A
R	1 ~ 13	13	N/A
U5	14 ~ 127	114	N/A
gag	262 ~ 2097	1836	611
pro	1932 ~ 2,858	927	308
pol	2,825 ~ 5,437	2,613	870
orf-x	4,602 ~ 5,066	465	154
env	5,346 ~ 7,193	1848	615
U3	7,175 ~ 7,441	267	N/A
TATA_box	7,421 ~ 7,428	8	N/A
Ploy A	7,436 ~ 7,441	6	N/A
R	7,442 ~ 7,454	13	N/A

LTR, long terminal repeat; N/A, not applicable.

#### 3.3.1 Whole genome similarity analysis

Comparative analysis revealed low sequence diversity between NMJS12 and other exJSRV isolates, with the highest identity (98.8%) to the Chinese strain JSRV-C1. Compared to JSRV-C1, NMJS12 exhibited six nucleotide mutations in its non-coding region (U3 region of the LTR); however, the coding regions contained 31 amino acid mutations spanning the LTR, Gag, Pro, Pol, Orf-x, and Env proteins (Supplementary Table S1). Compared to isolates outside China, sequence identity ranged from 93.05 to 95.84%, indicating the highest similarity (95.84%) to the French strain, JSRV FR1298 FR, and the lowest (93.05%) to multiple French-derived strains. Identity to enJSRV reference strains ranged from 88.73 to 92.26% (Supplementary Table S2).

#### 3.3.2 Key areas enabling primary distinction between enJSRV and exJSRV

##### 3.3.2.1 LTR

**Figure 3:**
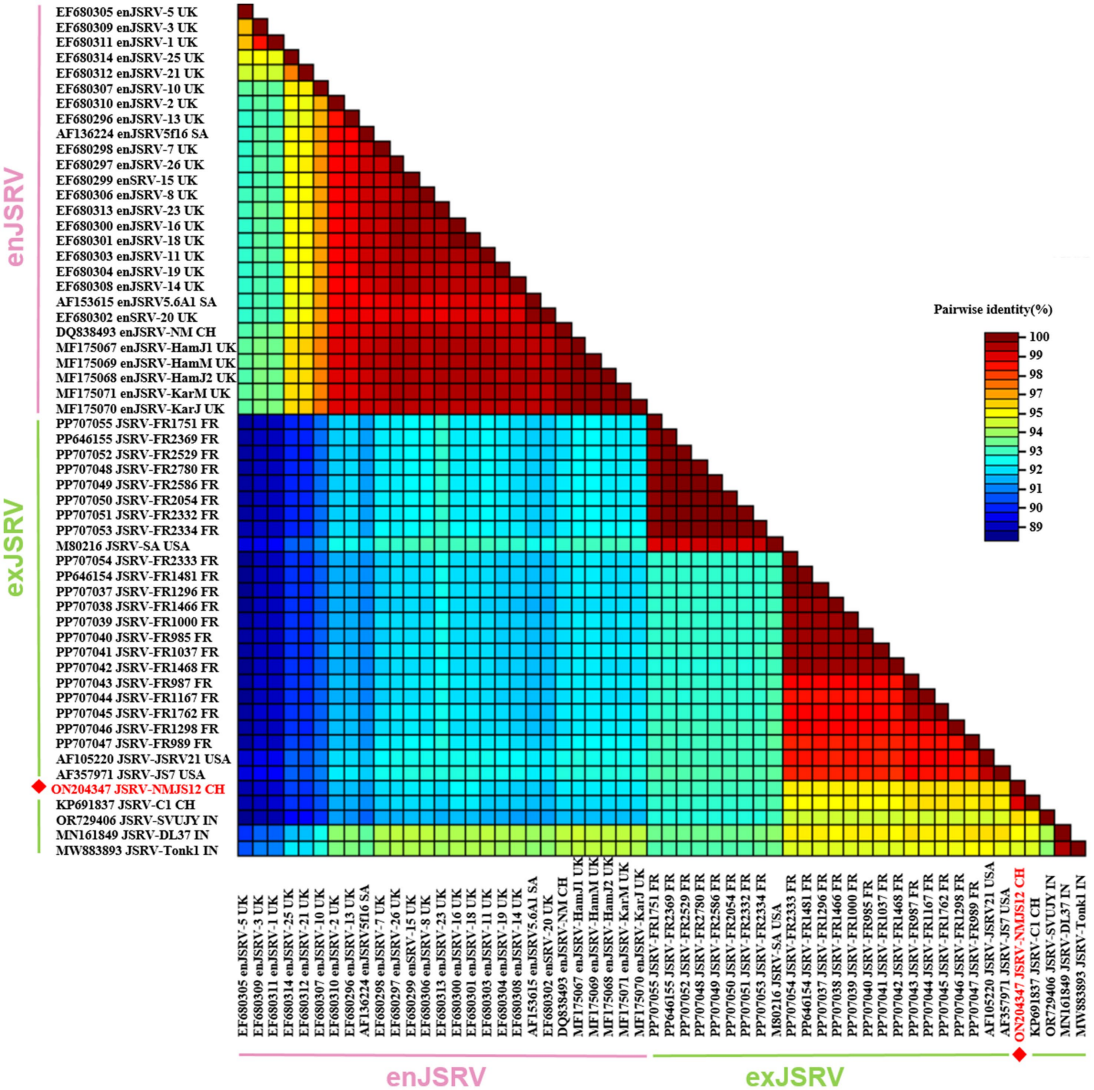
Pairwise whole-genome identity matrix of exJSRV and enJSRV. The NMJS12 genome amplified in this study is marked with a red diamond.

**Figure 4:**
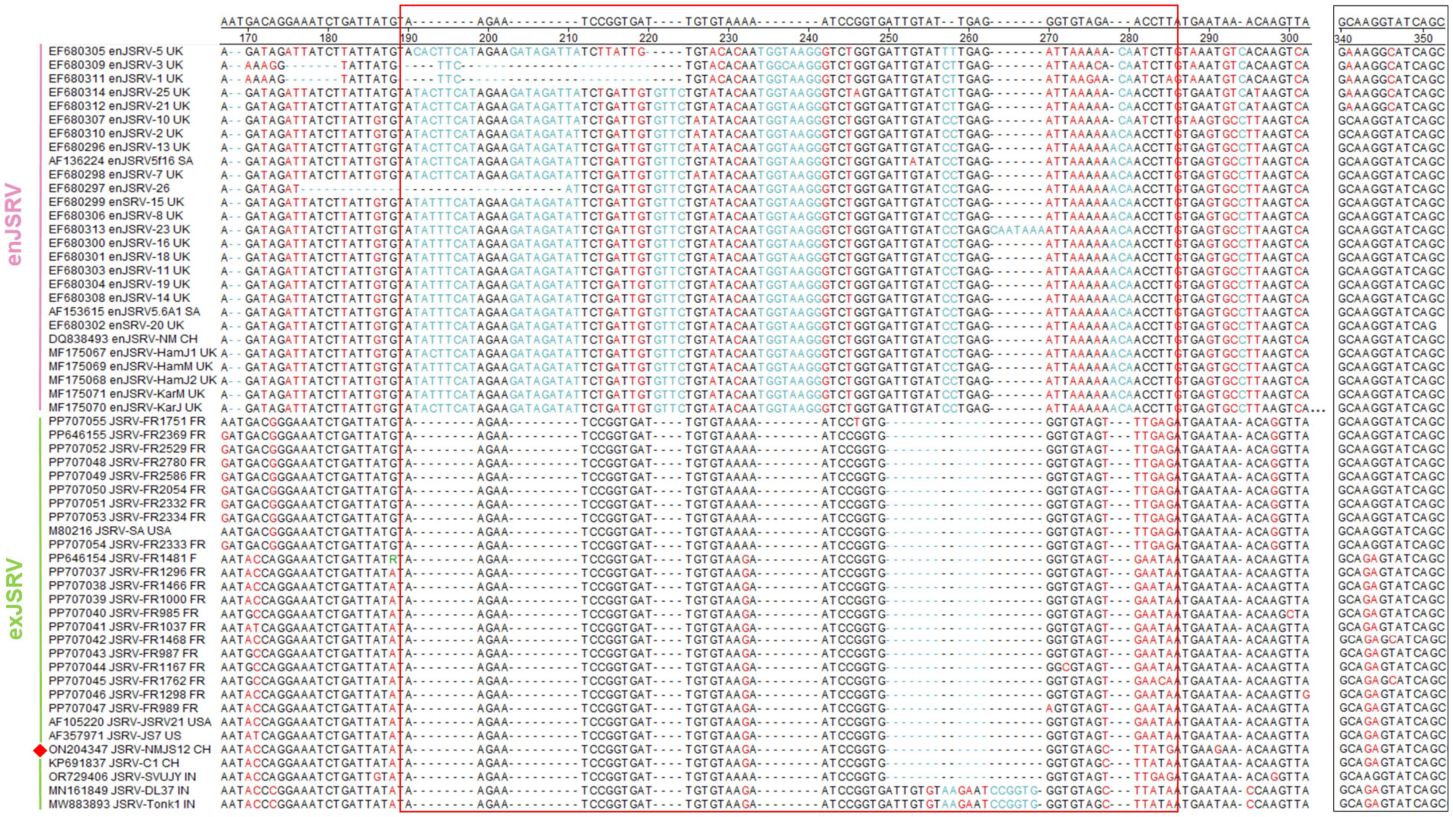
Regions of significant divergence in LTR sequences between enJSRV and exJSRV. The red boxes indicate the divergent regions while black boxes denote the R region. Sequence mutations are depicted in red while sequence insertions are colored blue. The NMJS12 genome amplified in this study is marked with a red diamond.

Similar to other retroviruses, the LTR of strain NMJS12 spans 392 bp (nucleotides 7,175–7,566) and comprises the U3 (267 bp), R (13 bp), and U5 (112 bp) regions (Table 2). Sequence identity to other exJSRV strains ranged from 90.28 to 98.98%, while nucleotide identity with enJSRV isolates ranged from 79.47 to 85% (Supplementary Table S3, Figure 3). Comparative analysis revealed high conservation in the R and U5 regions between endogenous viruses and exogenous exJSRV; however, significant divergence was observed in the U3 region. The U3 region harbors cis-acting elements critical for viral replication and transcription regulatory signals, including a TATA box and a poly A signal located at positions −1 to −21. The primary divergence in the LTR between the NMJS12 strain, other exJSRV isolates, and enJSRV was localized to nucleotides −91 to −60 within the U3 region (Figure 4, red box). In this region, the endogenous loci exhibited multiple insertion fragments: 8 nucleotide insertions at positions −91 to −90, 9 nucleotide insertions at −86 to −85, 4 nucleotide insertions at −78 to −77, 8 nucleotide insertions at −69 to −68, and 13 nucleotide insertions at −61 to −60, all of which were highly conserved in the exJSRV strains (including NMJS12). In contrast, the Indian strains, JSRV-DL37 and JSRV-Tonk1, displayed a distinct 19 amino acid (aa) insertion at positions −61 to −60. Notably, despite the high sequence identity in the LTR-R region between enJSRV and exJSRV (indicated by a black box), Clustal W alignment revealed distinct mutational patterns. Compared to other endogenous strains (e.g., enJSRV-3 and enJSRV-18), enJSRV-1, enJSRV-5, enJSRV-21, and enJSRV-25 exhibited stable G to A and T to C mutations at positions +2 and +7 within the R region, respectively. Similarly, the exogenous strains, FR1762 and FR1468, displayed a T-to-C mutation at position +7 in the R region (reference strain: JSRV-NMJS12).

##### 3.3.2.2 Gag

**Figure 5:**
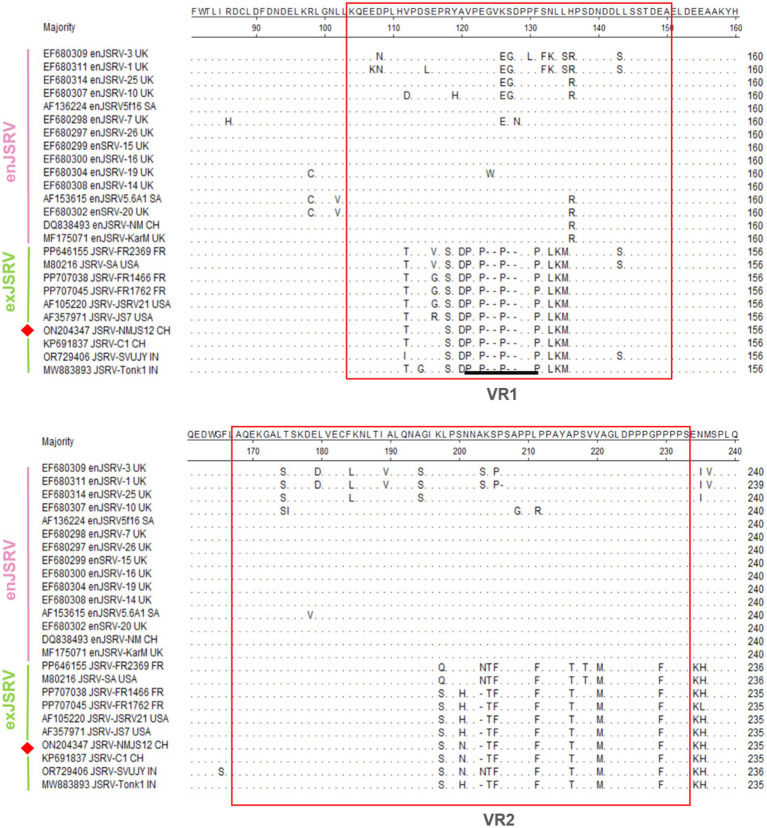
Alignment of the gag amino acid sequence (a representative strain was arbitrarily selected from sequences with 100% amino acid identity to simplify the alignment presentation). The red box represents VR1 and VR2; dots indicate identical sequences while dashes indicate lack of sequence. Black underlined section represents the differences in proline residues and position. The NMJS12 genome amplified in this study is marked with a red diamond.

**Figure 6:**
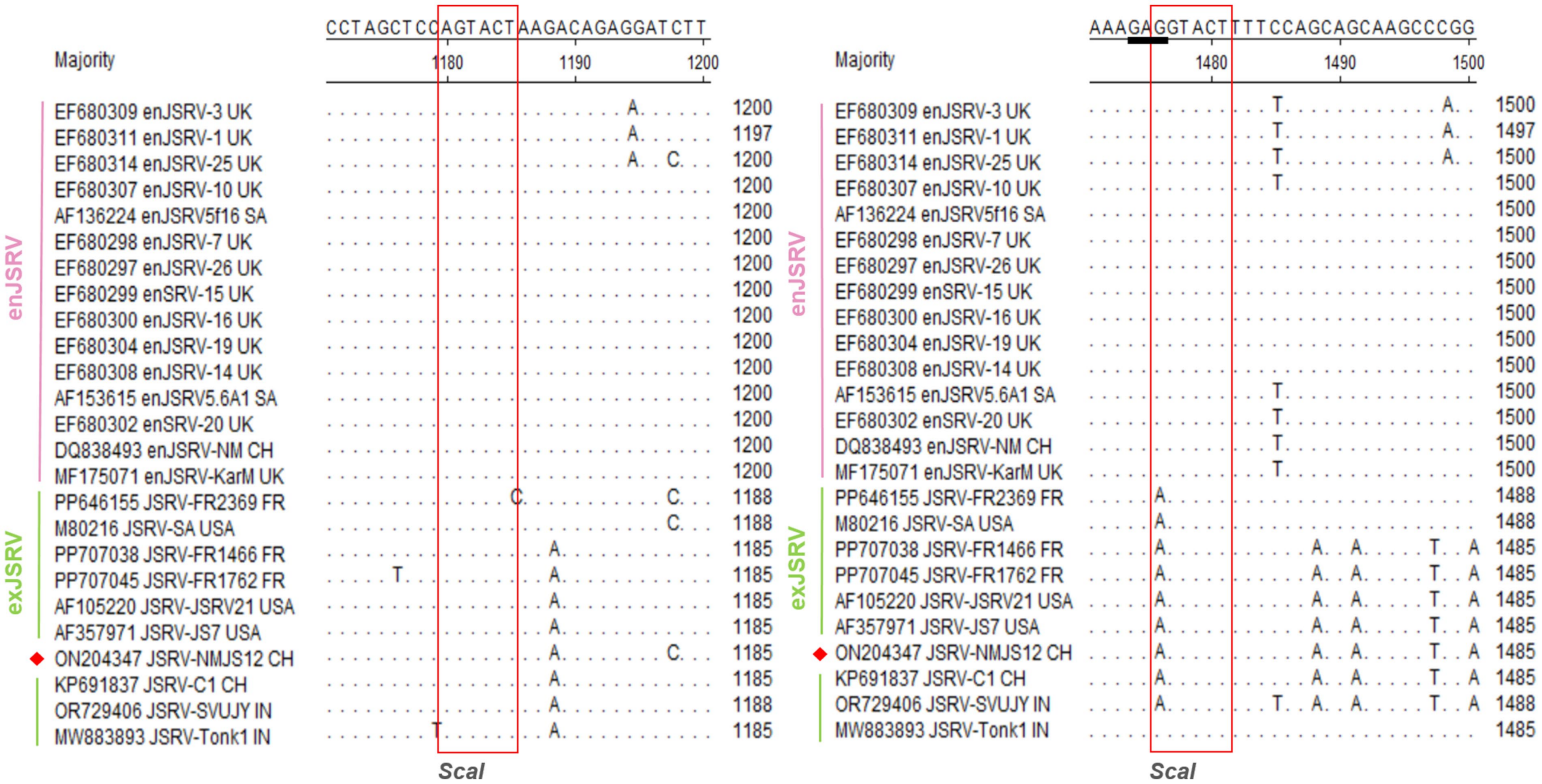
Alignment of the gag nucleotide sequence (a representative strain was arbitrarily selected from sequences with 100% amino acid identity to simplify the alignment presentation). Dashes indicate lack of sequence. The red box represents the restriction site, *ScaI.* The black bold underline represents the codons encoding Glu (E). The NMJS12 genome amplified in this study is marked with a red diamond.

The gag gene of NMJS12 spans 1,836 nt and encodes a polypeptide with a predicted molecular weight of 68 kDa. Clustal W alignment of the Gag amino acid sequences revealed 98.03–99.51% overall identity to other exJSRV strains and 92.96–95.74% similarity to enJSRV (Supplementary Table S4). The most pronounced divergence was found to localize to the previously described VR1 region (Figure 5), where exJSRV contains seven consecutive proline (Pro) residues. Another region of notable divergence from enJSRV was VR2 in gag, characterized by a limited number of amino acid mutations. Two canonical zinc finger motifs (Cys-X2 Cys-X4-His-X4-Cys), corresponding to sequences 508–521 (CFVCGQPGHRAAVC) and 535–548 (CPRCKKGKHWARDC), are predicted to mediate nucleocapsid genomic RNA binding (23, 24). Notably, the gag of exJSRV contains two highly conserved *ScaI* restriction sites separated by approximately 302 nucleotides, with the second *ScaI* site serving as a JSRV-specific marker. However, the French strain, JSRV FR2369, retains only the downstream *ScaI* site. The restriction sites *ScaI* of NMJS12 are located at 1165–1170 nt and 1,461–1,466 nt(GATACT), with the same specific marker of exJSRV. All enJSRV gags have only one restriction site, *ScaI* (the position is consistent with that of exJSRV 1,165–1,170 nt) (Figure 6). Additionally, the amino acid encoded by the 1,459–1,461 nt of exJSRV is ‘E’ (the key codon encoding Glu (E) is as indicated by the black bold underline in Figure 6), which is similar to the amino acid aligned with enJSRV.

##### 3.3.2.3 Pro

**Figure 7:**
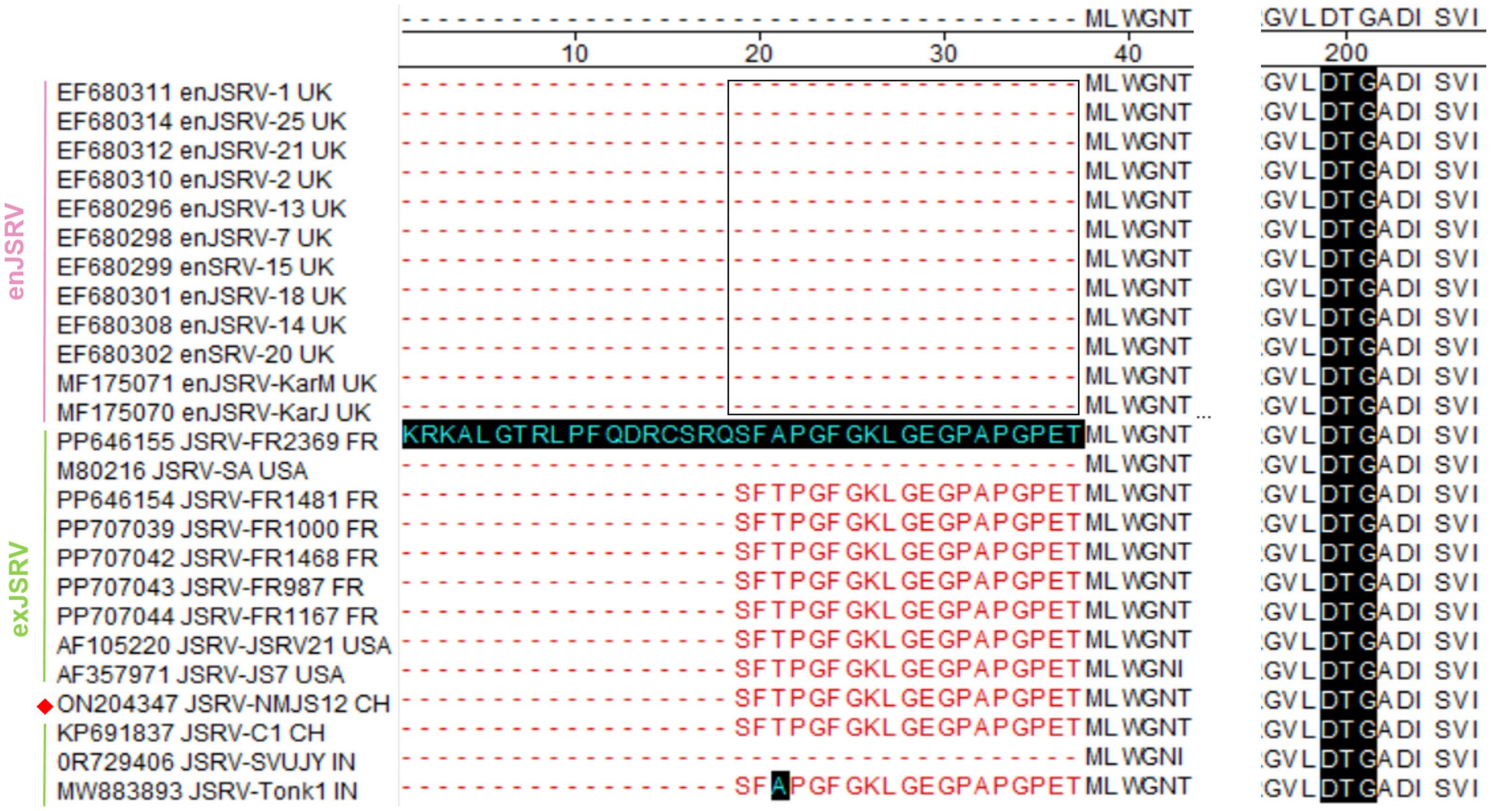
Alignment of the pro amino acid sequence (a representative strain was arbitrarily selected from sequences with 100% amino acid identity to simplify the alignment presentation). Dashes indicate lack of sequence. The black bar represents the DTG active site of the viral protease. Blue letters on a black bar background denote exJSRV strains exhibiting amino acid mutations. The NMJS12 genome amplified in this study is marked with a red diamond.

The pro gene encodes a 308-amino acid polypeptide (predicted molecular weight: 33 kDa), with its product comprising an N-terminal deoxyuridine triphosphatase (dUTPase) and a C-terminal viral protease. Notably, compared to enJSRV, the translation initiation site of the pol gene in most exJSRV strains is shifted 19 amino acids upstream, corresponding to the conserved sequence, SFTPGFGKLGEGPAPGPET (Figure 7). Starting from the 20th amino acid residue, the Pro protein of the NMJS12 strain exhibited 97.58 to 100% amino acid similarity relative to other exJSRV strains, indicating near-complete conservation; Sequence identity to enJSRV variants was 94.81–99.65% (Supplementary Table S5). Notably, despite a phylogenetic relationship between NMJS12 and the Chinese strain, JSRV-C1, their amino acid sequence identity was only 98.7%, which is slightly lower than that observed with other exJSRV strains. The 3′-terminal region of the pro gene is predicted to encode protease functionality. The DTG active site of the viral protease, a highly conserved feature among most retroviruses, spans nucleotide positions 2,469–2,495 (corresponding to positions 2,472–2,480 nt within the pro-encoded protein of NMJS12).

##### 3.3.2.4 Pol

**Figure 8:**
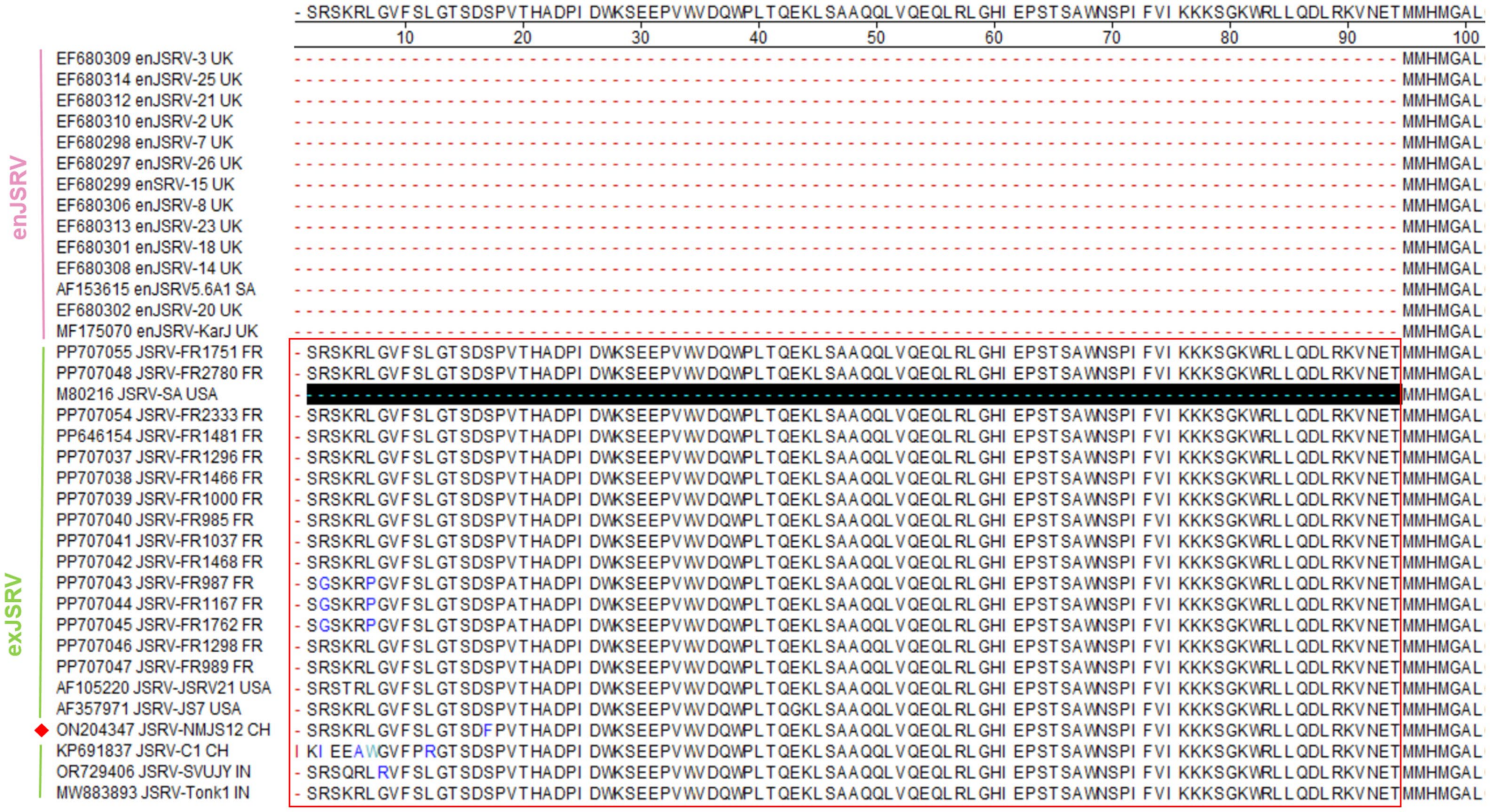
Alignment of the Pol amino acid sequence (a representative strain was arbitrarily selected from sequences with 100% amino acid identity to simplify the alignment presentation). Dashes indicate lack of sequence. Blue letters on a black bar background denote exJSRV strains exhibiting amino acid mutations. Red boxes indicate the conserved regions of exJSRV. The NMJS12 genome amplified in this study is marked with a red diamond.

The ORF of the pol gene encodes a viral reverse transcriptase and integrase and comprises an 870 aa polypeptide with a predicted molecular weight of 99 kDa. In NMJS12, the pol region spans nucleotides 2,825–5,437. Based on comparative amino acid sequence alignment with other retroviruses, the translation initiation site of the pro gene in NMJS12 and most other exJSRV strains is shifted approximately 93–94 amino acids upstream relative to enJSRV (highlighted by the red box in Figure 8), except that of JSRV-SA (indicated by the black bar in Figure 8). Notably, only few exJSRV strains exhibit amino acid mutations within this region. Starting from position 94 (95), the Pol protein of the NMJS12 isolate has high identity to other exJSRV strains, with amino acid identity ranging from 97.36 to 98.28%. The sequence identity to enJSRV variants also ranges from 94.34 to 98.33% (Supplementary Table S6).

##### 3.3.2.5 Orf-x

**Figure 9:**
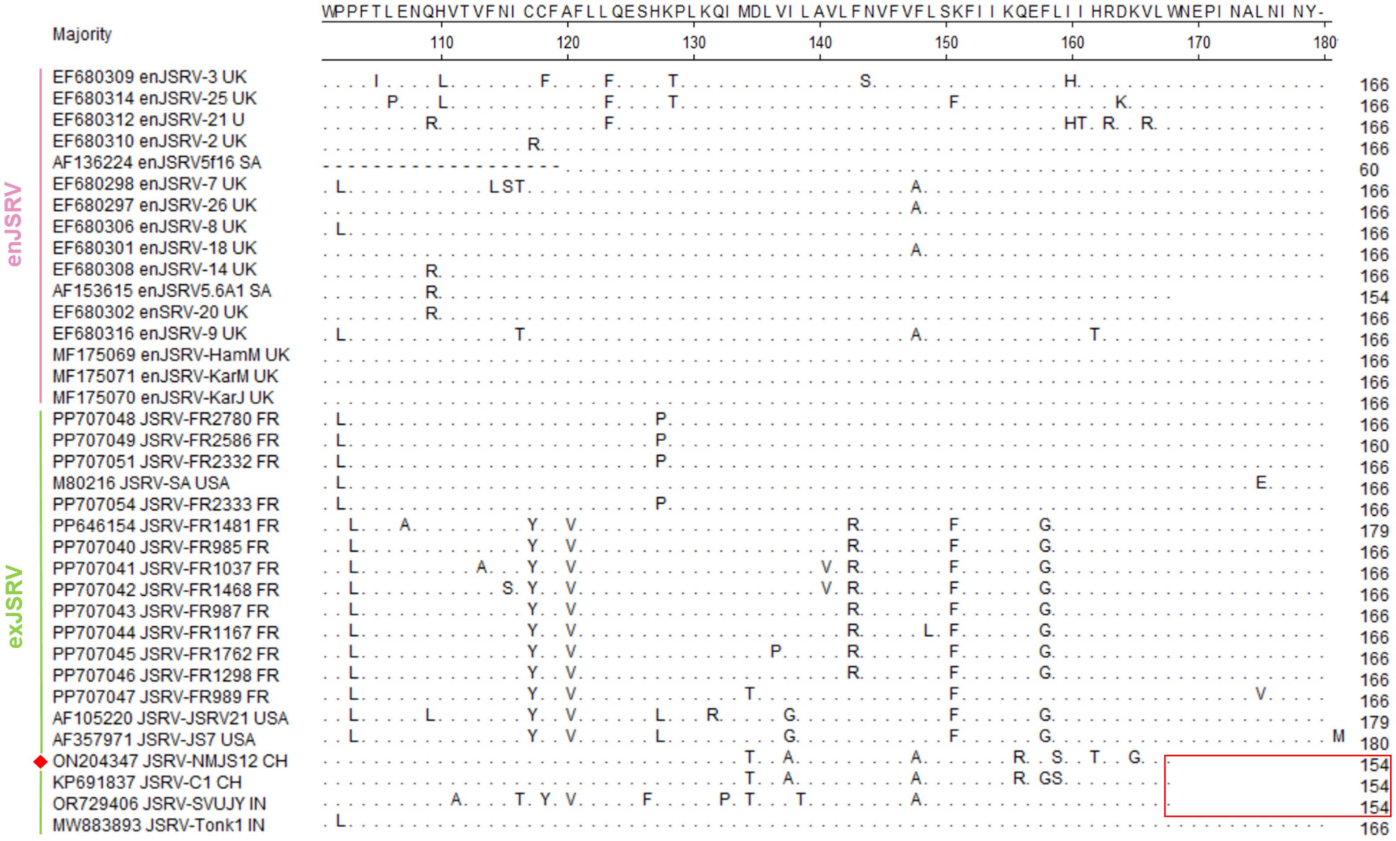
Alignment of the Orf-x amino acid sequence (a representative strain was arbitrarily selected from sequences with 100% amino acid identity to simplify the alignment presentation). Red boxes indicate the early translation termination pattern. The NMJS12 genome amplified in this study is marked with a red diamond.

The orf-x ORF of the NMJS12 strain encodes a 154-amino acid polypeptide chain (predicted molecular weight: ~17.7 kDa) nested within the pol gene; however, its codon usage pattern differs significantly from that of the pol coding sequence. Clustal W comparison showed that early termination of translation of Orf-x amino acid sequences (caused by premature in-frame stop codons) was only observed in Chinese strains NMJS12 and JSRV-C1, Indian strains JSRV-SUVJY, and some enJSRV sequences (Figure 9) (25). Amino acid similarity analysis showed that NMJS12 had the highest similarity with JSRV-C1, and the similarity range with other exJSRV/enJSRV strains was 83.12–96.75% and 79.22–90.26%, respectively (Supplementary Table S7).

##### 3.3.2.6 Env

**Figure 10:**
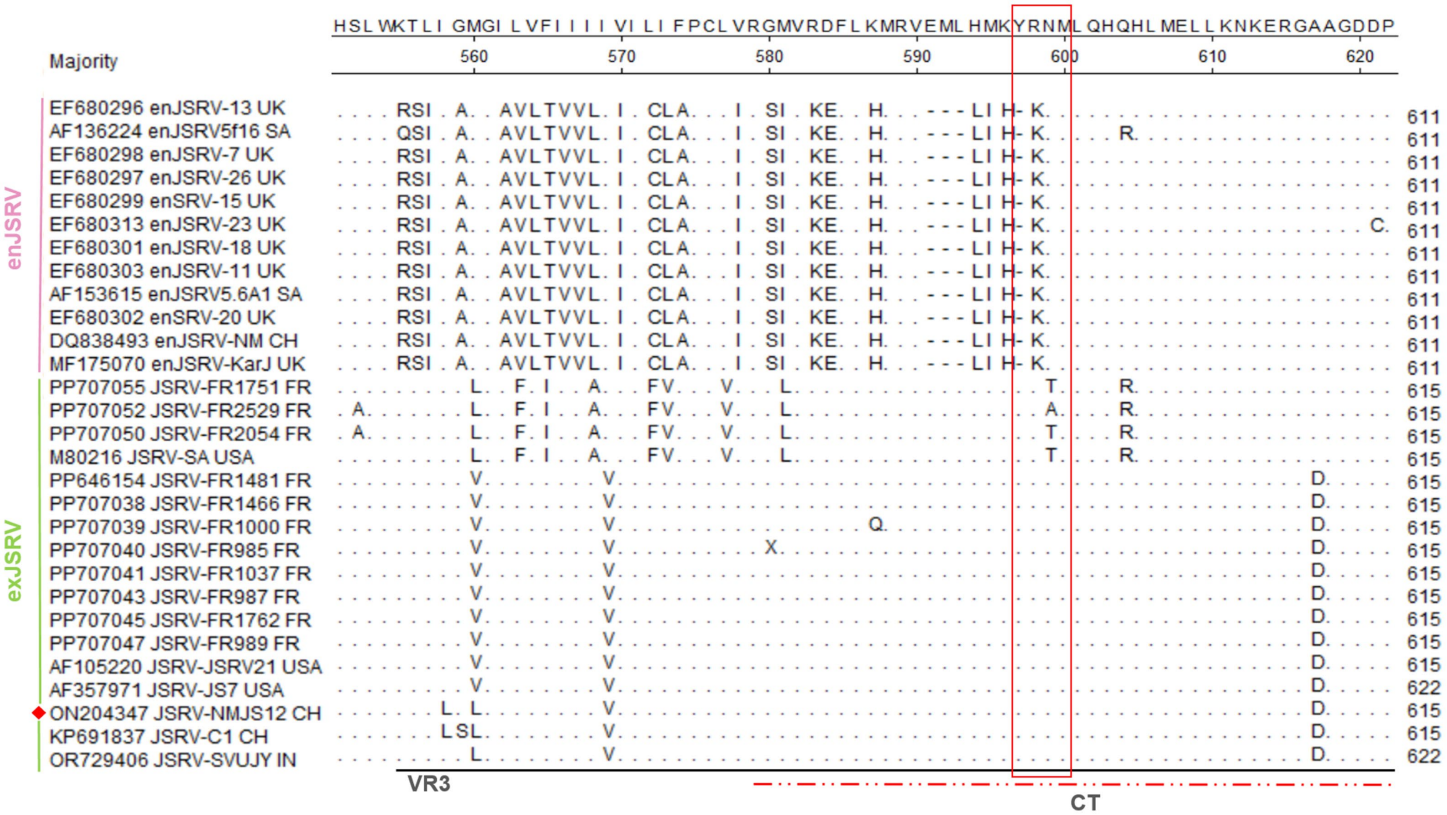
Alignment of the Env amino acid sequence (a representative strain was arbitrarily selected from sequences with 100% amino acid identity to simplify the alignment presentation). Dots indicate identical sequences while dashes indicate lack of sequence. The YXXM motif is outlined with red box. The NMJS12 genome amplified in this study is marked with a red diamond.

The env gene (nucleotide 5,346–7,193) of the NMJS12 strain encodes a 615-amino acid envelope glycoprotein with a predicted molecular weight of 69.4 kDa. The glycoprotein is cleaved by host intracellular protease at the highly conserved RPKP↓GLS motif (Furin Cleavage Motif) to generate the N-terminal surface (SU, amino acid positions 1,378) and a C-terminal TM domains (amino acid positions 379–615) (3). As shown in Figure 10, Clustal W sequence alignment revealed that the env gene translation start codon of JSRV-JS7 and JSRV-SVUJY was shifted 7 amino acids upstream compared to other retroviruses, but the latter (original start site) was confirmed to be a functional start codon (26). The exJSRV Env protein differs very little at the amino acid sequence level, with NMJJS12 sharing 97.40–99.67% similarity with other exJSRV strains and 91.98–92.96% identity with enJSRV variants (Supplementary Table S8). Functional partition analysis showed that enJSRV and exJSRV had high variation in variable region 3 (VR3) of cytoplasmic tail (CT) region: exJSRV contained tyrosine residues (Y590/597) in this region, while enJSRV retained equally conserved histidine (H586) within its CT domain.

### 3.4 Phylogenetic analyses

**Figure 11:**
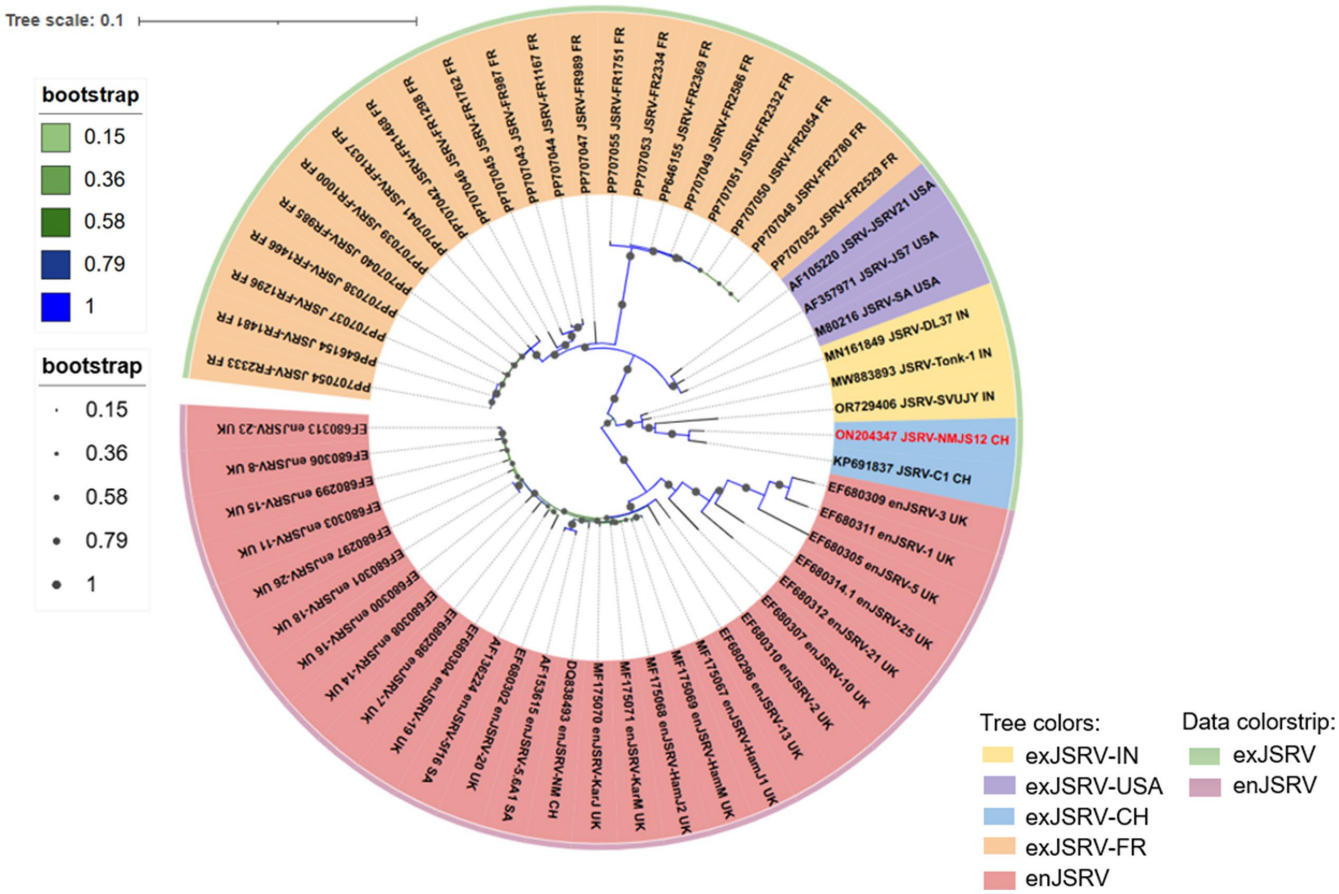
Phylogenetic analysis. Phylogenetic analysis of the evolutionary relationship between full-length nucleotides of enJSRV and exJSRV. The exJSRV strain, NMJS12, isolated in this study is highlighted in red.

The phylogenetic tree constructed by the neighbor-joining method revealed two primary clades: exJSRV (bootstrap value 0.769) and enJSRV (bootstrap value 1). The exJSRV clade further differentiated into two subclades, one containing the Indian strains Tonk-1 and DL37 (bootstrap value 1), and the other encompassing strains from China, the United States, and France (bootstrap value 1). It is particularly noteworthy that the Xinjiang strain from China and NMJS12 from this study clustered within the same high support subclade (bootstrap value 1), with an extremely close genetic distance (nucleotide difference <2%) indicating descent from a recent common ancestor (Figure 11). Notably, these two Chinese strains and the Indian strain SVUJY formed a further subclade (bootstrap value 0.999), suggesting a potential common evolutionary origin or historical transmission between Chinese and Indian strains. Additionally, another subclade resolved into three further subclades; among these, JSRV-SA, annotated as a US strain in GenBank, was confirmed through genomic tracing to be of South African origin. Its clustering with some French strains indicates a shared ancestor, and despite the vast geographical distance, transmission may have occurred via routes such as legal trade of breeding stock and circulation of biological products. Meanwhile, the Gag and Env protein trees were consistent with the full-length analysis, and the endogenous/exogenous clustering results were consistent (Supplementary Figures S1, S5), whereas the Pro, Pol, and Orf-x protein trees exhibited significantly different topologies (Supplementary Figures S2–S4).

### 3.5 Construction of the pc-NMJS12 clone

**Figure 12:**
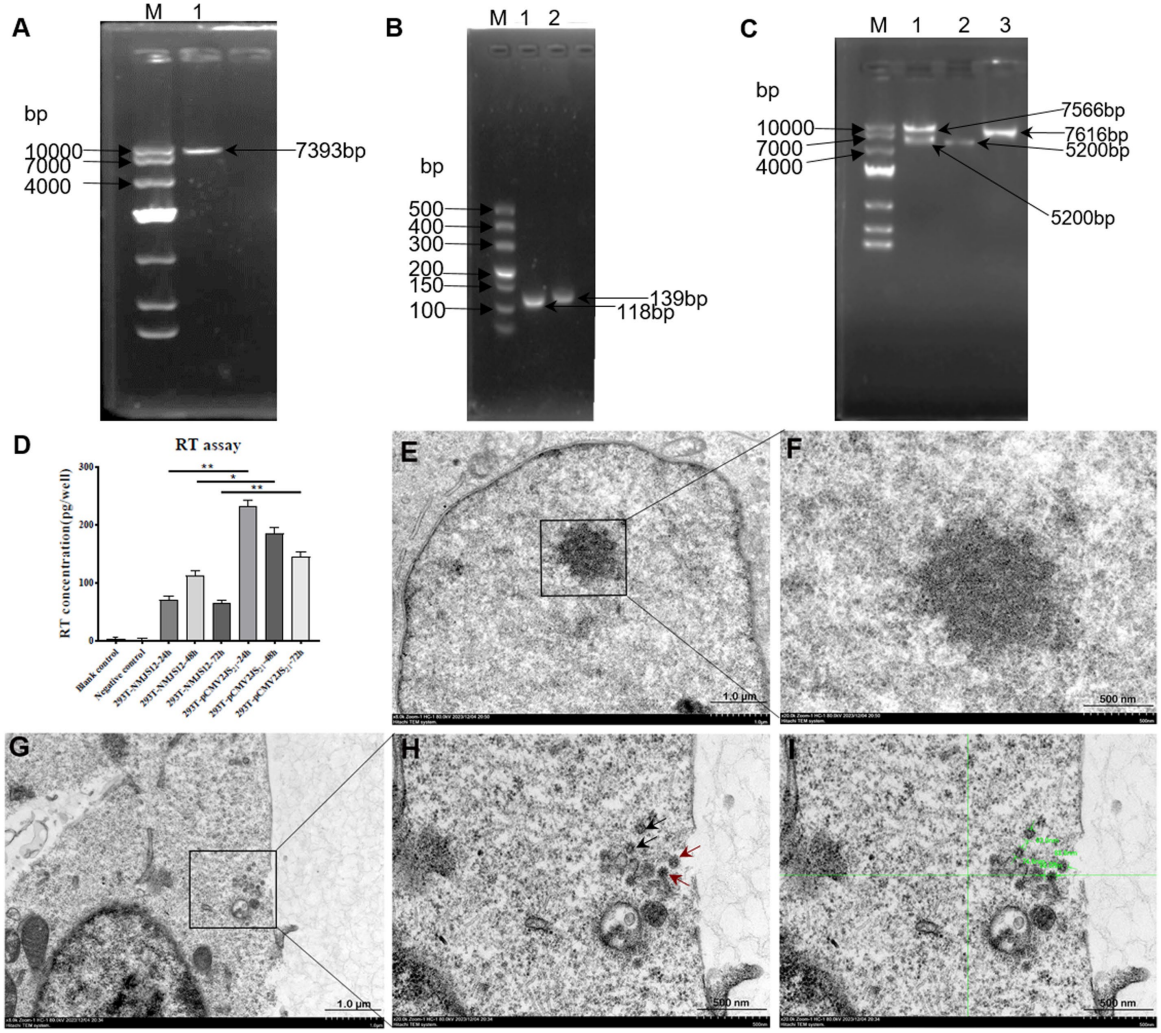
Agarose gel electrophoresis image of the full-length molecular cloning construction of JSRV-NMJS12. **(A)** PCR product from pc-NMJS12-*KpnI*-F/U3u-NMJS12-R; lane 1, the template consisted of PCR products amplified from JSRV-infected sheep lung tissues using NMJSRV-F/NMJSRV-R primers. **(B)** PCR product from U3d-F/U3d-U5-R (lane 1) and U3-U5-F/pc-U5-*NotI*-R (lane 2); the template consisted of PCR products amplified from exJSRV-infected sheep lung tissues using 3′RACE-F/3′RACE-R primers. **(C)** Restriction analysis of JSRV-NMJS12 molecular clone. *KpnI*/*NotI* digestion of JSRV-NMJS12 clone (lane 1); *KpnI*/*NotI*-digested pcDNA4.0 vector (lane 2); PCR product amplified with primers pc-NMJS12-*KpnI*-F and pc-U5-*NotI*-R (lane 3). **(D)** Reverse transcriptase assay. Comparative analysis of RT activity levels revealed statistically significant differences (*p < 0.05, **p < 0.01; Student’s *t*-test) between the 293T-NMJS12 group and 239T-pCMV2JS21 group. No detectable RT activity was observed between the blank control group (293T cell-only sample) and negative control group (pcDNA 4.0-only sample), confirming the absence of nonspecific enzymatic interference. **(E–I)** TEM images of viral particles produced by exJSRV plasmid transfection in 293T cells (scale bars: 100 nm); **(E, F)** Untreated 293T cells (no plasmid transfection); **(G–I)** Particles displaying typical spherical enveloped structures with electron-dense cores; Scale bars represent 1.0 μm **(E, G)** or 500 nm **(F, H, I)**.

Based on the existing research foundation, a redesigned primer with a homologous arm was successfully amplified to obtain three target fragments of 7,593 bp, 118 bp, and 129 bp (Figures 12A,B). The three amplified products were cloned into the eukaryotic expression vector pcDNA4.0 by *fusion PCR (homologous recombination)* and T4 DNA ligase (Figure 12C). The U3 region of the 5′-terminal LTR was replaced by the strong promoter of human cytomegalovirus (CMV) to drive high gene expression. The pc-NMJS12 whole genome clone was successfully constructed for viral particle packing (Figure 2B).

### 3.6 RT activity of rescued viruses

The target gene was cloned into a eukaryotic expression vector containing the human CMV immediate-early promoter. Using pc-NMJS12 and pCMV2JS21 (serving as a positive control) (1), 293T cells were transfected respectively; culture supernatants were collected at 24, 48, and 72 h post-transfection. The supernatants were centrifuged at 12,000 × *g* for 10 min to remove cellular debris, and 50 μL aliquots were used for RT activity assays, with three replicates per sample set to ensure result accuracy.

The results showed detectable viral RT activity at all time points. The positive control pCMV2JS21 exhibited average RT activities of 232.6, 185, and 145.3 pg/well at 24, 48, and 72 h, respectively, with the highest activity at 24 h. In contrast, NMJS12 displayed average RT activities of 71.7, 113.7, and 66.3 pg/well at the corresponding time points, its peak activity occurring at 48 h. Importantly, the activity of NMJS12 at all tested time points was significantly lower than that of the American reference strain JSRV21 (Figure 12D).

### 3.7 Visualization of virus particles

Since no *in vitro* culture system existed, the virions were successfully packaged, as demonstrated using transmission electron microscopy (TEM). TEM imaging and morphological analysis were performed on the purified NMJS12 virions. Compared with the blank control group (Figures 12E,F), complete round particles with a diameter of 75–89 nm can be seen in Figures 12G,H (box-like region) (Figure 12I). The core electron density (red arrow) was higher than that of the intracytoplasmic particles (black arrow), confirming the complete capsid structure and indicating budding from plasma membrane, in line with the budding mechanism of virus particles (27).

## 4 Discussion

The construction of full-length exogenous JSRV proviral molecular clones faces significant technical challenges due to interference from enJSRV. Researchers led by Palmarini successfully isolated a full-length exJSRV proviral clone (pJSRV₂₁, GenBank accession number: AF105220) using genomic DNA derived from OPA tissue. This isolation was achieved through *Xba I* restriction enzyme digestion followed by U3 hn-PCR combined with *ScaI* restriction site screening (1, 28). The De Martini group also amplified an exJSRV proviral DNA clone (AF357971) by constructing a λDash II phage genomic library from a sheep-derived pulmonary tumor cell line (JS7) (29).

This study successfully isolated a retroviral particle layer through density gradient centrifugation technology (buoyant density range 1.16–1.18 g/mL). However, the RT activity in ultracentrifugation-concentrated lesion lung tissue homogenate was significantly lower than that in MVV-infected cell supernatant, which might be attributed to exJSRV viral activity attenuation caused by prolonged frozen storage of pathological lung samples. A dual validation system was established for preliminary purification of exJSRV virions: First, reverse transcriptase activity detection (with no RT activity detected in corresponding density fractions of healthy control lung tissues), combined with U3 region-specific nested PCR identification, constructed a reliable technical pathway for viral particle purification.

Notably, enJSRV is highly expressed in the reproductive system, and its transcript product has also been detected in lung bronchial epithelial cells (30). To eliminate enJSRVs homologous sequence insertion, based on the genomic sequence analysis of exogenous exJSRV reference strains (AF105220 and AF357971), we designed specific reverse transcription primers in the low identity regions of U3 region, gag VR1/VR2 and env VR3 gene. The exJSRV-specific cDNA was obtained by targeted reverse transcription, and the near full-length viral genome was amplified using long-fragment high-fidelity PCR. The poly(A) tail site of viral RNA was amplified by 3′ RACE (31). The complete genome sequence of NMJS12 strain in Inner Mongolia was finally analysed (GenBank accession number: ON204347). Phylogenetic analysis showed that the genetic differences between enJSRV and exJSRV mainly existed in the U3 region of LTR, the variable region of capsid protein encoded by gag gene (VR1/VR2), and the variable region of envelope protein encoded by env gene (VR3). The NMJS12 strain in this study showed typical molecular characteristics of exJSRV in the above key regions. The LTR of exJSRV is the core regulatory element of viral transcription, and its U3 region contains HNF-3, NF-I, and C/EBP transcription factor binding sites. These determine the tropism of the virus to lung epithelial cells and are key molecular markers that distinguish exJSRV from enJSRV (1, 32, 33). The LTR U3 region of the Inner Mongolia-derived strain NMJS12 in this study was highly conserved with the reference exJSRV. However, we observed small nucleotide sequence variations (Figure 4), which may be due to differences in geographic distribution. The potential impact of these variants on viral packaging efficiency needs to be further verified using reverse genetics experiments.

The gag gene primarily encodes structural proteins, and its variable regions (VR1 and VR2) are located in the matrix protein (MA) domain. The salient feature of exJSRV is that the VR1 region contains a heptaproline motif (PPPPPPPS), which is absent in enJSRV (34). This study confirmed that the VR1/VR2 sequence of NMJS12 is consistent with the characteristics of exJSRV. In addition, two SCAI-limiting sites (1,165–1,170 nt, 1,461–1,466 nt) are present in the genome of exJSRV, whereas enJSRV retains only 1,461–1,466 nt sites, and this difference has been used for proviral library screening (1, 7). Although the French strain JSRV-FR2369 carries a single *ScaI* restriction site (phenotypic similar to endogenous retrovirus enJSRV), it fully retains the molecular characteristics of typical exJSRV; however, this strain had a synonym mutation (C → T) at 1,165–1,170 nt, resulting in loss of restriction function. This molecular feature suggests the need to integrate multimolecular features in the identification of exogenous viruses to improve the reliability of differential diagnosis. The *ScaI* restriction site pattern of NMJS12 was consistent with that of other exJSRV, further supporting its exogenous viral properties.

ExJSRV is a unique oncogenic retrovirus, because the capsule glycoprotein (Env) encoded by its env gene is a major oncogenic key structural protein, and its variable region VR3 is located in the TM protein domain (aa 572–615, corresponding to genomic position nt 1,714–1,845), which is one of the primary genomic differences between exJSRV and enJSRV (35). In this study, we found that the sequence of Env protein VR3 region of the Inner Mongolia isolate NMJS12 is highly similar to the exJSRV reference strain, but significantly different from enJSRVs, which is consistent with previously reported results. Notably, exJSRV Env proteins can induce malignant transformation of NIH3T3 and other cells through the conflated “YXXM” motif of their TM region CT, which is dependent on tyrosine (Y590) phosphorylation in the motif and its mediated activation of PI3K/Akt signaling pathway (36). Benjamin et al. recently published epidemiological data from multiple regions in France over a 20-year period showing that strains carrying the “YRTM” motif (T592 replaces wild type N592) are associated with an increased incidence of OPA in sheep; however, the pathogenesis remains unlucidated (37). The Env protein of NMJS12 isolated in this study has a “YXXM” motif (Y590-R591-N592-M593) and exhibits motif conservation with *β* retrovirus members such as enzootic nasal tumor virus (ENTV), suggesting genetic stability of “YXXM” (23).

Phylogenetic analysis revealed significant differences between the endogenous and exogenous virus strains, and the NMJS12 strain of Inner Mongolia was clustered in the evolutionary branch of exJSRV strains, closely related to JSRV-C1 of Xinjiang strain in China. The Indian strain (SVUJY) is more closely related to JSRV-C1 than NMJS12, which may be related to the international import and export of animals. The significant sequence difference between NMJS12 and SVUJY suggested that the molecular adaptive evolutionary mechanism of the virus was possible. In addition, phylogenetic analysis of the Inner Mongolia and the Xinjiang strains indicated that the common ancestor of the virus had a relatively recent differentiation, suggesting that the virus may have evolved conservatively in China.

In this study, the pc-NMJS12 molecular clone was successfully constructed based on preliminary research. Because of the lack of *in vitro* culture system, we referred to the construction strategy of JSRV21 infectious molecular clone established by Palmarini et al. (1), leveraging homologous recombination to replace the upstream U3 regulatory region with the CMV immediate-early promoter. TEM analysis following transient transfection of 293T cells revealed that the pc-NMJS12 molecular clone could package exJSRV virions, with viral particles exhibiting pleomorphic characteristics and observable budding events. Notably, the RT activity of NMJS12 strain was significantly lower than that of JSRV21 reference strain (*p < 0.05, **p < 0.01), suggesting functional differences among different geographical strains. However, the correlation between RT activity and virus titer has not been fully established and should be explored in future studies. Based on these findings, we plan to perform animal infection experiments, mainly to explore the following: (1) *in vivo* infectivity of NMJS12 strain; (2) the pathogenicity difference between NMJS12 and JSRV21 strains; and (3) molecular mechanisms underlying phenotypic variations. The aim is to explore the adaptive evolutionary mechanism of exJSRV and the correlation between regional differences and its pathogenicity.

To our knowledge, this study is the first to systematically characterise a full-genome molecular clone of exJSRV from Inner Mongolia (GenBank accession: ON204347) and confirm high conservation of critical molecular features, including LTR regulatory elements (U3), gag variable regions, and env oncogenic motifs, with exJSRV. The cross-regional transmission clues of Chinese strain JSRV-C1 and Indian strain SVUJY were revealed by comparison of genomic sequences, which provided basic data for molecular epidemiological studies of exJSRV. The establishment of the pc-NMJS12 reverse genetics system provides a technical foundation for future exploration of viral geographical adaptive evolution mechanisms and potential pathogenicity differences.

## 5 Conclusion

This study revealed through regional evolutionary analysis that the Inner Mongolia strain NMJS12 shares a close genetic relationship with the Xinjiang strain JSRV-C1, suggesting that exJSRV may exhibit potential cross-regional transmission or co-evolution characteristics between adjacent areas such as Inner Mongolia and Xinjiang. Based on these findings, the constructed pc-NMJS2 molecular clone successfully achieved *in vitro* viral packaging, preliminarily confirming the biological activity of the NMJS12 strain and laying the groundwork for subsequent infectious molecular clones. This study provides the first systematic characterization of key molecular features of regional viral strains, establishing a data foundation for developing highly sensitive diagnostic methods (e.g., PCR assays targeting specific gene fragments) for exJSRV. In the future, integrating sequence data with epidemiological investigations could enhance the monitoring and management of OPA in Inner Mongolia. Potential applications include molecular tracing of viral transmission routes and implementing region-specific control measures, thereby mitigating economic losses in the livestock industry.

## Data Availability

The datasets presented in this study can be found in online repositories. The names of the repository/repositories and accession number(s) can be found in the article/Supplementary material.

## Footnotes

^1^ https://www.megasoftware.net/
