# Supplementary material for: Exogenous Jaagsiekte sheep retrovirus (JSRV) Inner Mongolia strain: whole-genome characterization and viral particle packaging
Source: Front Vet Sci. 2025 Aug 18;12:1608822. doi: 10.3389/fvets.2025.1608822 (PMC12400676; doi:10.3389/fvets.2025.1608822)
Supplement: Supplementary file 1 [file Presentation_1.PPTX]

## Slide 1
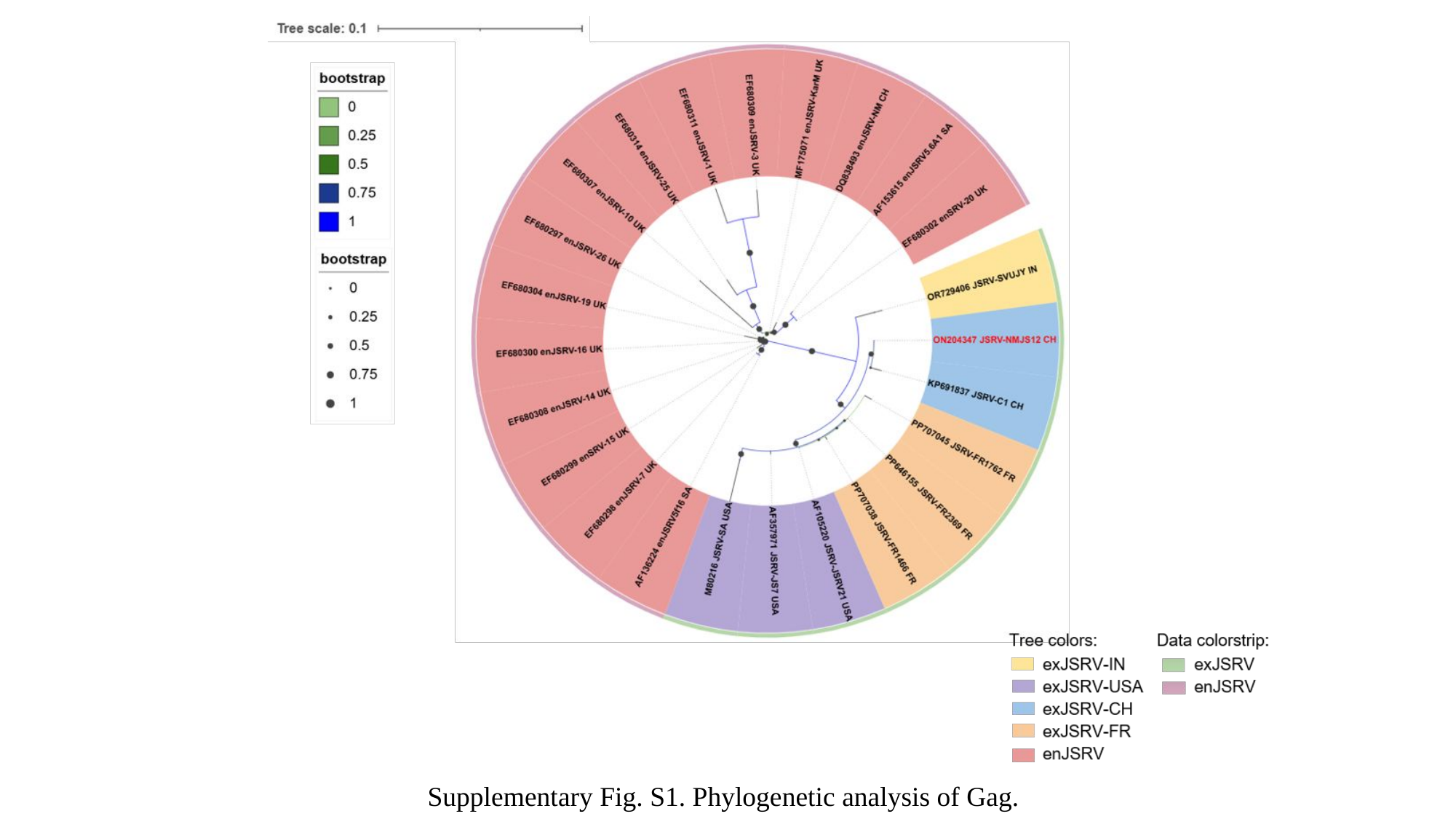

Supplementary Fig. S1. Phylogenetic analysis of Gag.

## Slide 2
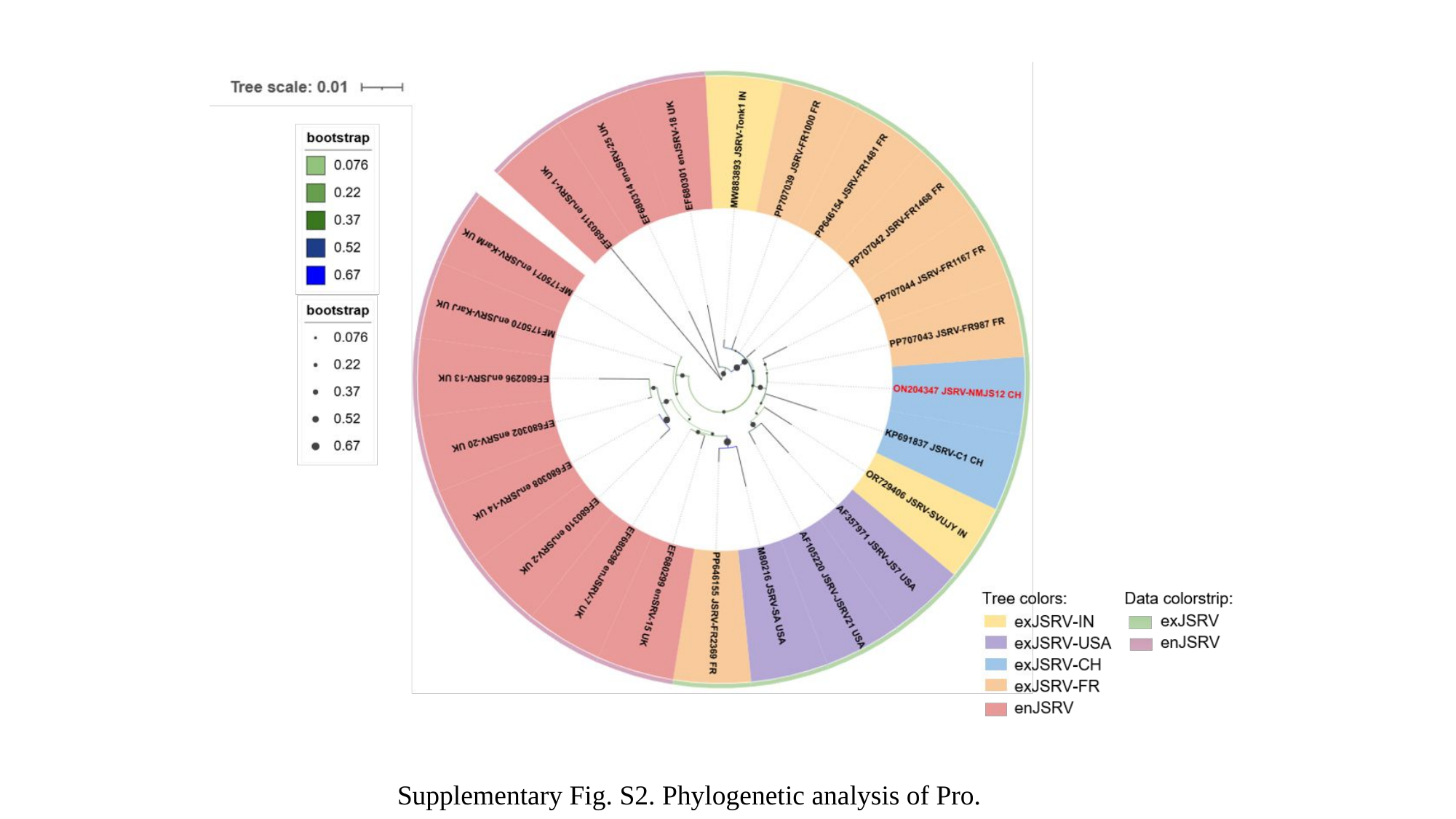

Supplementary Fig. S2. Phylogenetic analysis of Pro.

## Slide 3
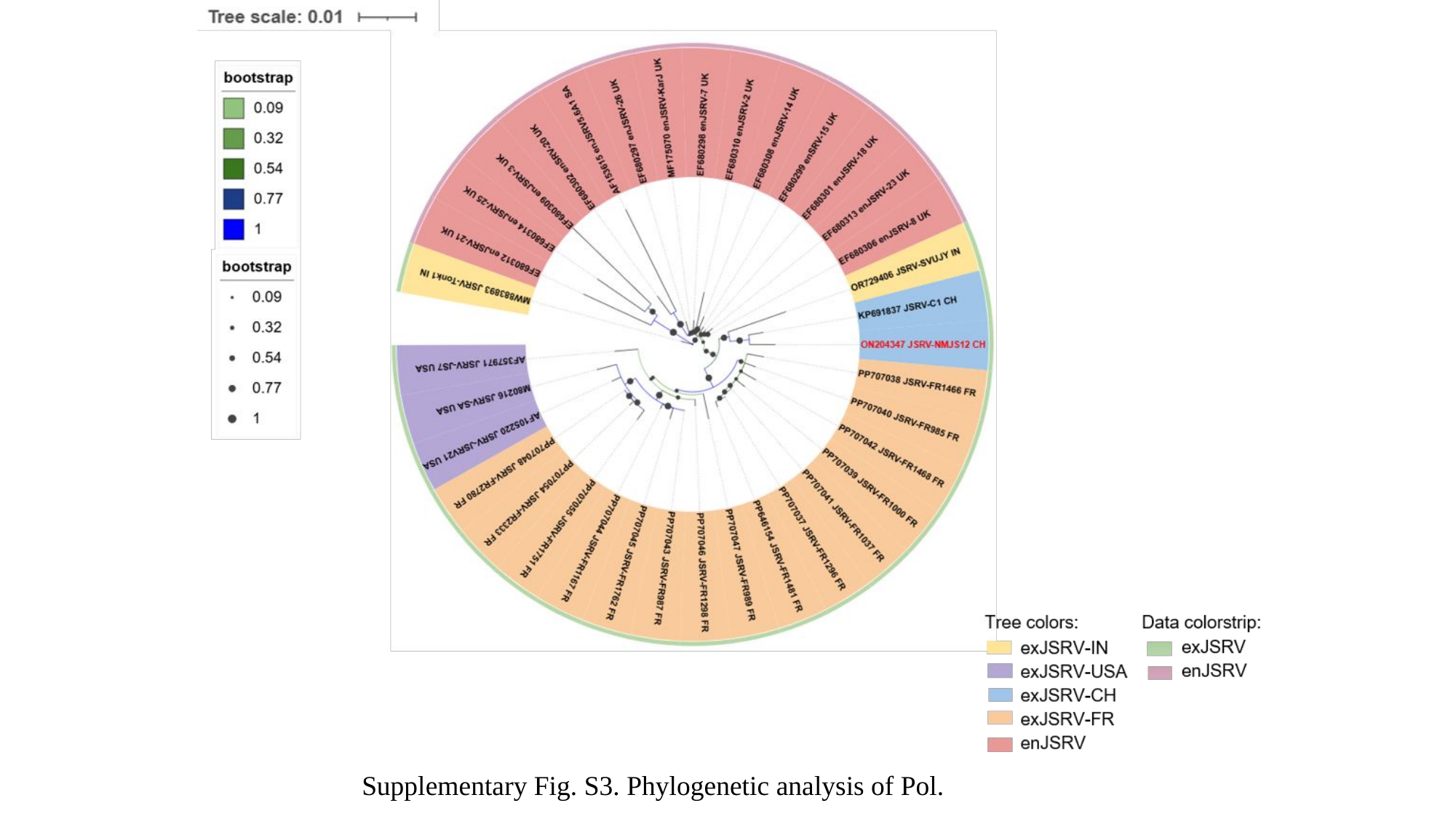

Supplementary Fig. S3. Phylogenetic analysis of Pol.

## Slide 4
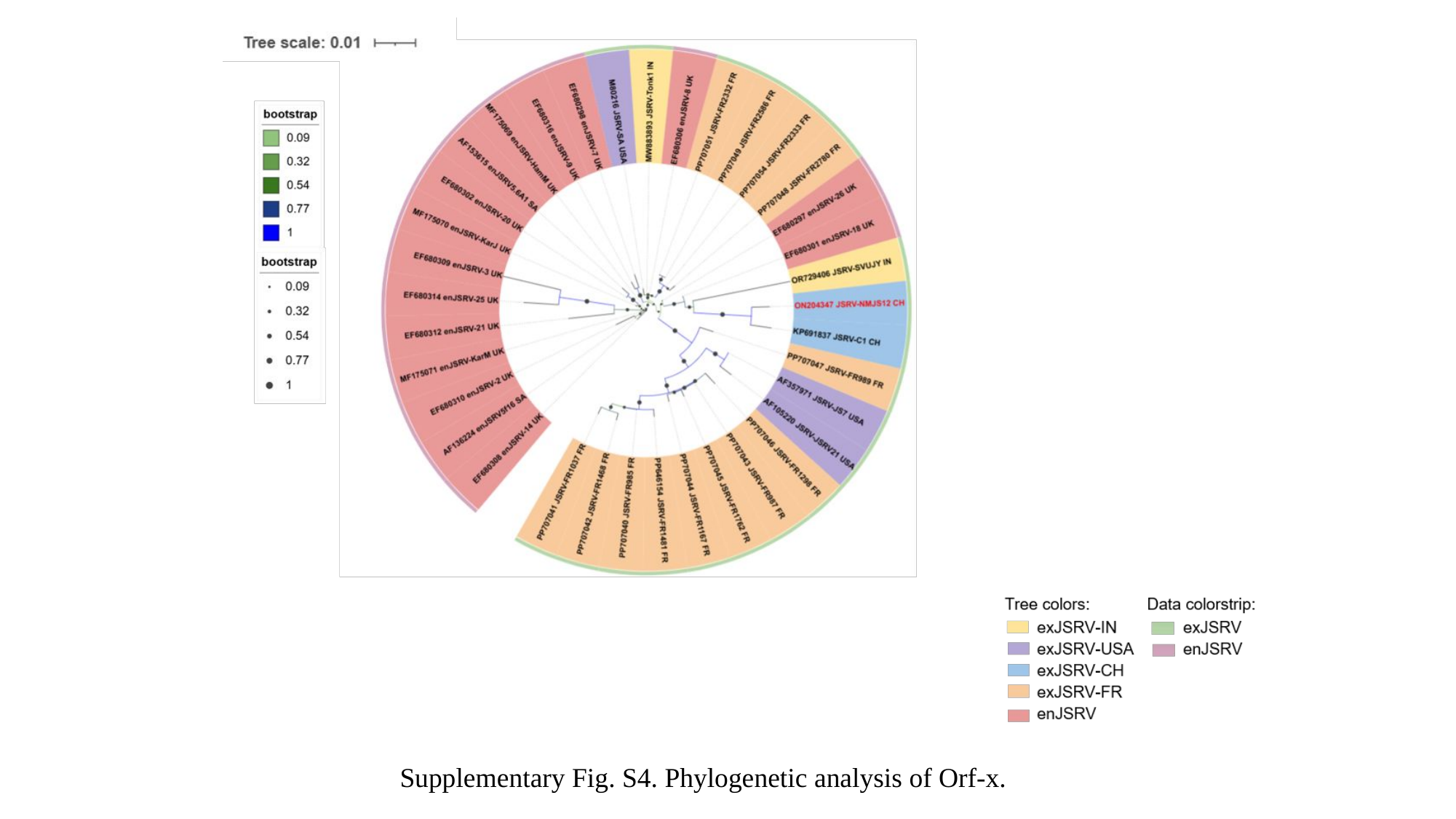

Supplementary Fig. S4. Phylogenetic analysis of Orf-x.

## Slide 5
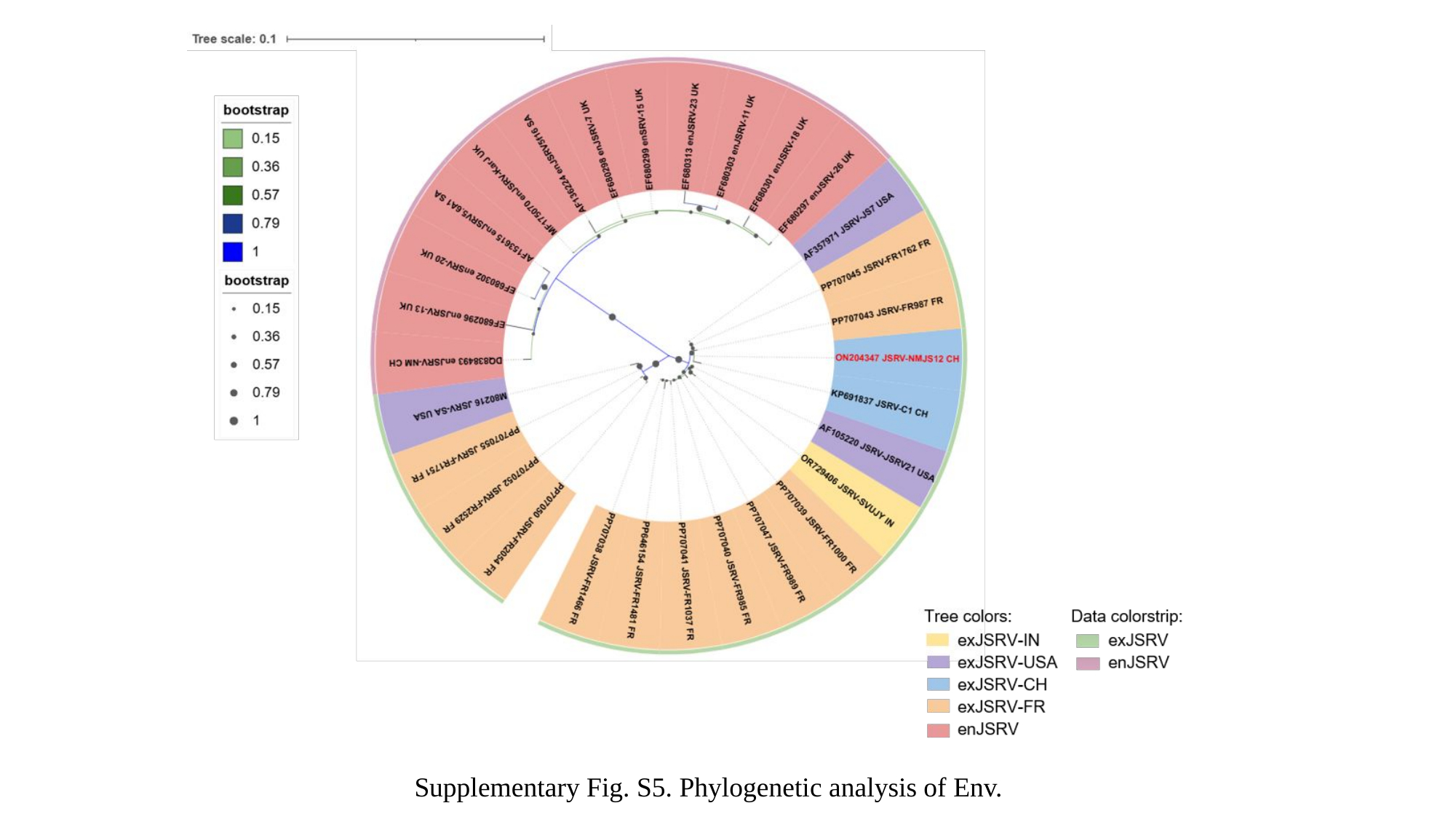

Supplementary Fig. S5. Phylogenetic analysis of Env.
