# Supplementary material for: Exogenous Jaagsiekte sheep retrovirus (JSRV) Inner Mongolia strain: whole-genome characterization and viral particle packaging
Source: Front Vet Sci. 2025 Aug 18;12:1608822. doi: 10.3389/fvets.2025.1608822 (PMC12400676; doi:10.3389/fvets.2025.1608822)
Supplement: Supplementary file 2 [file Table_1.DOCX]

**Table S1.** Mutation sites of nucleotide and amino acid between Chinese Jaagsiekte Sheep Retrovirus isolates

| **Name(LTR and Protein)** | **Mutation position** | JSRV-NMJS12 | JSRV-C1 |
| --- | --- | --- | --- |
| LTR | 1 | - | G |
|  | 2 | - | A |
|  | 22 | A | G |
|  | 135 | T | C |
|  | 222 | G | A |
|  | 228 | G | T |
| Gag | 43 | W | R |
|  | 270 | P | L |
|  | 468 | K | R |
| Pro | 95 | P | S |
|  | 120 | N | D |
|  | 289 | I | V |
|  | 302 | K | R |
| Pol | 1 | - | I |
|  | 2 | S | K |
|  | 3 | R | I |
|  | 4 | S | E |
|  | 5 | K | E |
|  | 6 | R | A |
|  | 7 | L | W |
|  | 10 | S | P |
|  | 11 | L | R |
|  | 16 | F | S |
|  | 203 | A | T |
|  | 230 | K | R |
|  | 763 | R | K |
|  | 808 | R | K |
|  | 848 | V | I |
|  | 870 | T | A |
|  | 872 | - | R |
|  | 873 | - | A |
|  | 874 | - | H |
|  | 875 | - | E |
| Orf-x | 16 | L | P |
|  | 69 | N | S |
|  | 144 | E | G |
|  | 148 | T | I |
|  | 151 | G | D |
| Env | 279 | N | S |
|  | 506 | S | G |

**Table S2.** Identity comparison of the whole genome (%) between NMJS12 and reference strains

| exJSRV | Similarity | enJSRV | Similarity |
| --- | --- | --- | --- |
| PP707042 JSRV-FR1468 FR | 0.9549299 | AF136224 enJSRV5f16 SA | 0.9113633 |
| PP707041 JSRV-FR1037 FR | 0.9553264 | AF153615 enJSRV5.6A1 SA | 0.9168986 |
| PP707047 JSRV-FR989 FR | 0.958102 | DQ838493 enJSRV-NM CH | 0.9203657 |
| PP707040 JSRV-FR985 FR | 0.9553264 | EF680296 enJSRV-13 UK | 0.9150431 |
| PP707038 JSRV-FR1466 FR | 0.9551943 | EF680297 enJSRV-26 UK | 0.9199151 |
| PP707039 JSRV-FR1000 FR | 0.955723 | EF680298 enJSRV-7 UK | 0.9197032 |
| PP707037 JSRV-FR1296 FR | 0.9553087 | EF680299 enSRV-15 UK | 0.9216908 |
| PP646155 JSRV-FR2369 FR | 0.9304601 | EF680300 enJSRV-16 UK | 0.9226183 |
| AF105220 JSRV-JSRV21 USA | 0.957601 | EF680301 enJSRV-18 UK | 0.9226183 |
| AF357971 JSRV-JS7 USA | 0.9575972 | EF680302 enSRV-20 UK | 0.9163906 |
| MW883893 JSRV-Tonk1 IN | 0.950599 | EF680303 enJSRV-11 UK | 0.9197032 |
| PP707053 JSRV-FR2334 FR | 0.9305372 | EF680306 enJSRV-8 UK | 0.9219558 |
| PP707052 JSRV-FR2529 FR | 0.9304601 | EF680308 enJSRV-14 UK | 0.9181132 |
| PP707051 JSRV-FR2332 FR | 0.9304601 | EF680310 enJSRV-2 UK | 0.914888 |
| PP707050 JSRV-FR2054 FR | 0.9304601 | EF680311 enJSRV-1 UK | 0.887264 |
| PP707049 JSRV-FR2586 FR | 0.9304601 | EF680313 enJSRV-23 UK | 0.9201124 |
| PP707048 JSRV-FR2780 FR | 0.9304601 | MF175067 enJSRV-HamJ1 UK | 0.9211607 |
| PP707046 JSRV-FR1298 FR | 0.9583664 | EF680314 enJSRV-25 UK | 0.9000974 |
| PP707045 JSRV-FR1762 FR | 0.9577055 | MF175068 enJSRV-HamJ2 UK | 0.9207632 |
| PP707044 JSRV-FR1167 FR | 0.957309 | MF175069 enJSRV-HamM UK | 0.9211607 |
| PP707043 JSRV-FR987 FR | 0.958102 | MF175070 enJSRV-KarJ UK | 0.9207632 |
| PP646154 JSRV-FR1481 FR | 0.9551765 | MF175071 enJSRV-KarM UK | 0.9210282 |
| OR729406 JSRV-SVUJY IN | 0.9582719 | EF680309 enJSRV-3 UK | 0.8873502 |
| KP691837 JSRV-C1 CH | 0.9877918 | EF680304 enJSRV-19 UK | 0.919 |
| M80216 JSRV-SA USA | 0.9334496 | EF680312 enJSRV-21 UK | 0.8984873 |
| PP707054 JSRV-FR2333 FR | 0.9305372 | EF680305 enJSRV-5 UK | 0.8836591 |
| PP707055 JSRV-FR1751 FR | 0.9304601 | EF680307 enJSRV-10 UK | 0.9034181 |
| MN161849 JSRV-DL37 IN | 0.9534258 | - | - |

**Table S3.** Identity comparison of the LTR (%) between NMJS12 and reference strains

| exJSRV | Identity | enJSRV | Identity |
| --- | --- | --- | --- |
| PP707042 JSRV-FR1468 FR | 0.9668368 | AF136224 enJSRV5f16 SA | 0.8363171 |
| PP707041 JSRV-FR1037 FR | 0.9668368 | AF153615 enJSRV5.6A1 SA | 0.8388747 |
| PP707047 JSRV-FR989 FR | 0.9617347 | DQ838493 enJSRV-NM CH | 0.8414322 |
| PP707040 JSRV-FR985 FR | 0.9668368 | EF680296 enJSRV-13 UK | 0.8388747 |
| PP707038 JSRV-FR1466 FR | 0.9693878 | EF680297 enJSRV-26 UK | 0.8393782 |
| PP707039 JSRV-FR1000 FR | 0.9668368 | EF680298 enJSRV-7 UK | 0.8363171 |
| PP707037 JSRV-FR1296 FR | 0.9693878 | EF680299 enSRV-15 UK | 0.8337596 |
| PP646155 JSRV-FR2369 FR | 0.9028133 | EF680300 enJSRV-16 UK | 0.8414322 |
| AF105220 JSRV-JSRV21 USA | 0.9745547 | EF680301 enJSRV-18 UK | 0.8414322 |
| AF357971 JSRV-JS7 USA | 0.9719388 | EF680302 enSRV-20 UK | 0.8414322 |
| MW883893 JSRV-Tonk1 IN | 0.9764398 | EF680303 enJSRV-11 UK | 0.8439898 |
| PP707053 JSRV-FR2334 FR | 0.9038961 | EF680306 enJSRV-8 UK | 0.8414322 |
| PP707052 JSRV-FR2529 FR | 0.9028133 | EF680308 enJSRV-14 UK | 0.8337596 |
| PP707051 JSRV-FR2332 FR | 0.9028133 | EF680310 enJSRV-2 UK | 0.8363171 |
| PP707050 JSRV-FR2054 FR | 0.9028133 | EF680311 enJSRV-1 UK | 0.8105263 |
| PP707049 JSRV-FR2586 FR | 0.9028133 | EF680313 enJSRV-23 UK | N/A |
| PP707048 JSRV-FR2780 FR | 0.9028133 | MF175067 enJSRV-HamJ1 UK | 0.8487179 |
| PP707046 JSRV-FR1298 FR | 0.9642857 | EF680314 enJSRV-25 UK | 0.8363171 |
| PP707045 JSRV-FR1762 FR | 0.9566327 | MF175068 enJSRV-HamJ2 UK | 0.8439898 |
| PP707044 JSRV-FR1167 FR | 0.9591837 | MF175069 enJSRV-HamM UK | 0.8465473 |
| PP707043 JSRV-FR987 FR | 0.9668368 | MF175070 enJSRV-KarJ UK | 0.8491049 |
| PP646154 JSRV-FR1481 FR | 0.9668368 | MF175071 enJSRV-KarM UK | 0.8439898 |
| OR729406 JSRV-SVUJY IN | 0.9491094 | EF680309 enJSRV-3 UK | 0.7947369 |
| KP691837 JSRV-C1 CH | 0.9898477 | EF680316 enJSRV-9 UK | 0.8465473 |
| M80216 JSRV-SA USA | 0.908397 | EF680317 enJSRV-4 UK | N/A |
| PP707054 JSRV-FR2333 FR | 0.9038961 | EF680312 enJSRV-21 UK | 0.831202 |
| PP707055 JSRV-FR1751 FR | 0.9053708 | EF680305 enJSRV-5 UK | 0.797954 |
| MN161849 JSRV-DL37 IN | 0.9753425 | EF680307 enJSRV-10 UK | 0.8414322 |

N/A, not applicable.

**Table.S4.** Compare the percentage of amino acid similarity between NMJS12 and the reference strain Gag.

| exJSRV | Similarity | enJSRV | Similarity |
| --- | --- | --- | --- |
| PP707042 JSRV-FR1468 FR | 0.9934534 | AF136224 enJSRV5f16 SA | 0.9574468 |
| PP707041 JSRV-FR1037 FR | 0.9934534 | AF153615 enJSRV5.6A1 SA | 0.9443535 |
| PP707047 JSRV-FR989 FR | 0.9934534 | DQ838493 enJSRV-NM CH | 0.9525368 |
| PP707040 JSRV-FR985 FR | 0.99509 | EF680296 enJSRV-13 UK | N/A |
| PP707038 JSRV-FR1466 FR | 0.9934534 | EF680297 enJSRV-26 UK | 0.9574468 |
| PP707039 JSRV-FR1000 FR | 0.99509 | EF680298 enJSRV-7 UK | 0.9574468 |
| PP707037 JSRV-FR1296 FR | 0.995082 | EF680299 enSRV-15 UK | 0.9558101 |
| PP646155 JSRV-FR2369 FR | 0.9819967 | EF680300 enJSRV-16 UK | 0.9574468 |
| AF105220 JSRV-JSRV21 USA | 0.99509 | EF680301 enJSRV-18 UK | 0.9574468 |
| AF357971 JSRV-JS7 USA | 0.99509 | EF680302 enSRV-20 UK | 0.9459902 |
| MW883893 JSRV-Tonk1 IN | 0.98527 | EF680303 enJSRV-11 UK | N/A |
| PP707053 JSRV-FR2334 FR | 0.9819967 | EF680306 enJSRV-8 UK | 0.9574468 |
| PP707052 JSRV-FR2529 FR | 0.9803601 | EF680308 enJSRV-14 UK | 0.9590835 |
| PP707051 JSRV-FR2332 FR | 0.9819967 | EF680310 enJSRV-2 UK | N/A |
| PP707050 JSRV-FR2054 FR | 0.9819967 | EF680311 enJSRV-1 UK | 0.9148936 |
| PP707049 JSRV-FR2586 FR | 0.9819967 | EF680313 enJSRV-23 UK | N/A |
| PP707048 JSRV-FR2780 FR | 0.9819967 | MF175067 enJSRV-HamJ1 UK | 0.9574468 |
| PP707046 JSRV-FR1298 FR | 0.9934534 | EF680314 enJSRV-25 UK | 0.9410802 |
| PP707045 JSRV-FR1762 FR | 0.9918167 | MF175068 enJSRV-HamJ2 UK | 0.9574468 |
| PP707044 JSRV-FR1167 FR | 0.9918167 | MF175069 enJSRV-HamM UK | 0.9574468 |
| PP707043 JSRV-FR987 FR | 0.99018 | MF175070 enJSRV-KarJ UK | 0.9574468 |
| PP646154 JSRV-FR1481 FR | 0.995082 | MF175071 enJSRV-KarM UK | 0.9574468 |
| OR729406 JSRV-SVUJY IN | 0.9934534 | EF680309 enJSRV-3 UK | 0.9198036 |
| KP691837 JSRV-C1 CH | 0.99509 | EF680316 enJSRV-9 UK | N/A |
| M80216 JSRV-SA USA | 0.9803601 | EF680317 enJSRV-4 UK | N/A |
| MN161849 JSRV-DL37 IN | 0.9918167 | EF680312 enJSRV-21 UK | N/A |
| PP707054 JSRV-FR2333 FR | 0.9819967 | EF680305 enJSRV-5 UK | N/A |
| PP707055 JSRV-FR1751 FR | 0.9819967 | EF680307 enJSRV-10 UK | 0.9296235 |

N/A, not applicable.

**Table.S5.** Compare the percentage of amino acid similarity between NMJS12 and the reference strain Pro.

| exJSRV | Similarity | enJSRV | Similarity |
| --- | --- | --- | --- |
| PP707042 JSRV-FR1468 FR | 0.9935065 | AF136224 enJSRV5f16 SA | 0.9896194 |
| PP707041 JSRV-FR1037 FR | 0.9967533 | AF153615 enJSRV5.6A1 SA | 0.9861591 |
| PP707047 JSRV-FR989 FR | 0.9935065 | DQ838493 enJSRV-NM CH | 0.9965398 |
| PP707040 JSRV-FR985 FR | 0.9967533 | EF680296 enJSRV-13 UK | 0.9723184 |
| PP707038 JSRV-FR1466 FR | 0.9967533 | EF680297 enJSRV-26 UK | 0.9930796 |
| PP707039 JSRV-FR1000 FR | 0.9935065 | EF680298 enJSRV-7 UK | 0.9896194 |
| PP707037 JSRV-FR1296 FR | 0.9967533 | EF680299 enSRV-15 UK | 0.9861591 |
| PP646155 JSRV-FR2369 FR | 0.9805195 | EF680300 enJSRV-16 UK | 0.9861591 |
| AF105220 JSRV-JSRV21 USA | 0.9935065 | EF680301 enJSRV-18 UK | 0.9861591 |
| AF357971 JSRV-JS7 USA | 0.987013 | EF680302 enSRV-20 UK | 0.9861591 |
| MW883893 JSRV-Tonk1 IN | 0.9902598 | EF680303 enJSRV-11 UK | 0.9930796 |
| PP707053 JSRV-FR2334 FR | 0.9805195 | EF680306 enJSRV-8 UK | 0.9896194 |
| PP707052 JSRV-FR2529 FR | 0.9805195 | EF680308 enJSRV-14 UK | 0.9861591 |
| PP707051 JSRV-FR2332 FR | 0.9805195 | EF680310 enJSRV-2 UK | 0.982699 |
| PP707050 JSRV-FR2054 FR | 0.9805195 | EF680311 enJSRV-1 UK | 0.9480969 |
| PP707049 JSRV-FR2586 FR | 0.9805195 | EF680313 enJSRV-23 UK | 0.9896194 |
| PP707048 JSRV-FR2780 FR | 0.9805195 | MF175067 enJSRV-HamJ1 UK | 0.9930796 |
| PP707046 JSRV-FR1298 FR | 1 | EF680314 enJSRV-25 UK | 0.9688581 |
| PP707045 JSRV-FR1762 FR | 1 | MF175068 enJSRV-HamJ2 UK | 0.9930796 |
| PP707044 JSRV-FR1167 FR | 0.9935065 | MF175069 enJSRV-HamM UK | 0.9930796 |
| PP707043 JSRV-FR987 FR | 1 | MF175070 enJSRV-KarJ UK | 0.9896194 |
| PP646154 JSRV-FR1481 FR | 0.9967533 | MF175071 enJSRV-KarM UK | 0.9930796 |
| OR729406 JSRV-SVUJY IN | 0.9896194 | EF680309 enJSRV-3 UK | N/A |
| KP691837 JSRV-C1 CH | 0.987013 | EF680316 enJSRV-9 UK | N/A |
| M80216 JSRV-SA USA | 0.9757785 | EF680317 enJSRV-4 UK | N/A |
| PP707054 JSRV-FR2333 FR | 0.9805195 | EF680312 enJSRV-21 UK | 0.9757785 |
| PP707055 JSRV-FR1751 FR | 0.9805195 | EF680305 enJSRV-5 UK | N/A |
| MN161849 JSRV-DL37 IN | N/A | EF680307 enJSRV-10 UK | N/A |

N/A, not applicable.

**Table.S6.** Compare the percentage of amino acid similarity between NMJS12 and the reference strain Pol.

| exJSRV | Similarity | enJSRV | Similarity |
| --- | --- | --- | --- |
| PP707042 JSRV-FR1468 FR | 0.9804597 | AF136224 enJSRV5f16 SA | N/A |
| PP707041 JSRV-FR1037 FR | 0.9804597 | AF153615 enJSRV5.6A1 SA | 0.9451697 |
| PP707047 JSRV-FR989 FR | 0.9781609 | DQ838493 enJSRV-NM CH | 0.983269 |
| PP707040 JSRV-FR985 FR | 0.9793103 | EF680296 enJSRV-13 UK | N/A |
| PP707038 JSRV-FR1466 FR | 0.9793103 | EF680297 enJSRV-26 UK | 0.980695 |
| PP707039 JSRV-FR1000 FR | 0.9793103 | EF680298 enJSRV-7 UK | 0.980695 |
| PP707037 JSRV-FR1296 FR | 0.9804597 | EF680299 enSRV-15 UK | 0.983269 |
| PP646155 JSRV-FR2369 FR | 0.9827586 | EF680300 enJSRV-16 UK | 0.983269 |
| AF105220 JSRV-JSRV21 USA | 0.9781609 | EF680301 enJSRV-18 UK | 0.983269 |
| AF357971 JSRV-JS7 USA | 0.976985 | EF680302 enSRV-20 UK | 0.972973 |
| MW883893 JSRV-Tonk1 IN | 0.9793103 | EF680303 enJSRV-11 UK | 0.983269 |
| PP707053 JSRV-FR2334 FR | 0.9816092 | EF680306 enJSRV-8 UK | 0.983269 |
| PP707052 JSRV-FR2529 FR | 0.9827586 | EF680308 enJSRV-14 UK | 0.979408 |
| PP707051 JSRV-FR2332 FR | 0.9827586 | EF680310 enJSRV-2 UK | 0.97426 |
| PP707050 JSRV-FR2054 FR | 0.9827586 | EF680311 enJSRV-1 UK | N/A |
| PP707049 JSRV-FR2586 FR | 0.9827586 | EF680313 enJSRV-23 UK | 0.981982 |
| PP707048 JSRV-FR2780 FR | 0.9827586 | MF175067 enJSRV-HamJ1 UK | 0.983269 |
| PP707046 JSRV-FR1298 FR | 0.9804597 | EF680314 enJSRV-25 UK | 0.956242 |
| PP707045 JSRV-FR1762 FR | 0.9735632 | MF175068 enJSRV-HamJ2 UK | 0.983269 |
| PP707044 JSRV-FR1167 FR | 0.9747127 | MF175069 enJSRV-HamM UK | 0.983269 |
| PP707043 JSRV-FR987 FR | 0.9758621 | MF175070 enJSRV-KarJ UK | 0.983269 |
| PP646154 JSRV-FR1481 FR | 0.9793103 | MF175071 enJSRV-KarM UK | 0.983269 |
| OR729406 JSRV-SVUJY IN | 0.9770115 | EF680309 enJSRV-3 UK | 0.943372 |
| KP691837 JSRV-C1 CH | 0.9816092 | EF680316 enJSRV-9 UK | N/A |
| M80216 JSRV-SA USA | 0.978121 | EF680317 enJSRV-4 UK | N/A |
| PP707054 JSRV-FR2333 FR | 0.9816092 | EF680312 enJSRV-21 UK | 0.9574742 |
| PP707055 JSRV-FR1751 FR | 0.9804597 | EF680305 enJSRV-5 UK | N/A |
| MN161849 JSRV-DL37 IN | N/A | EF680307 enJSRV-10 UK | N/A |

N/A, not applicable.

**Table.S7.** Compare the percentage of amino acid similarity between NMJS12 and the reference strain Orf-x.

| exJSRV | Similarity | enJSRV | Similarity |
| --- | --- | --- | --- |
| PP707042 JSRV-FR1468 FR | 0.8311688 | AF136224 enJSRV5f16 SA | 0.8541667 |
| PP707041 JSRV-FR1037 FR | 0.8311688 | AF153615 enJSRV5.6A1 SA | 0.8961039 |
| PP707047 JSRV-FR989 FR | 0.8896104 | DQ838493 enJSRV-NM CH | N/A |
| PP707040 JSRV-FR985 FR | 0.8441558 | EF680296 enJSRV-13 UK | N/A |
| PP707038 JSRV-FR1466 FR | 0.8441558 | EF680297 enJSRV-26 UK | 0.9155844 |
| PP707039 JSRV-FR1000 FR | 0.8441558 | EF680298 enJSRV-7 UK | 0.8831169 |
| PP707037 JSRV-FR1296 FR | 0.8376623 | EF680299 enSRV-15 UK | N/A |
| PP646155 JSRV-FR2369 FR | 0.8918919 | EF680300 enJSRV-16 UK | 0.9350649 |
| AF105220 JSRV-JSRV21 USA | 0.8571429 | EF680301 enJSRV-18 UK | 0.9350649 |
| AF357971 JSRV-JS7 USA | 0.8571429 | EF680302 enSRV-20 UK | 0.9025974 |
| MW883893 JSRV-Tonk1 IN | 0.9025974 | EF680303 enJSRV-11 UK | N/A |
| PP707053 JSRV-FR2334 FR | 0.8896104 | EF680306 enJSRV-8 UK | 0.9025974 |
| PP707052 JSRV-FR2529 FR | 0.8896104 | EF680308 enJSRV-14 UK | 0.8896104 |
| PP707051 JSRV-FR2332 FR | 0.8831169 | EF680310 enJSRV-2 UK | 0.8831169 |
| PP707050 JSRV-FR2054 FR | 0.8896104 | EF680311 enJSRV-1 UK | 0.9148936 |
| PP707049 JSRV-FR2586 FR | 0.8851351 | EF680313 enJSRV-23 UK | 0.9025974 |
| PP707048 JSRV-FR2780 FR | 0.8831169 | MF175067 enJSRV-HamJ1 UK | 0.8961039 |
| PP707046 JSRV-FR1298 FR | 0.8441558 | EF680314 enJSRV-25 UK | 0.8116883 |
| PP707045 JSRV-FR1762 FR | 0.8506494 | MF175068 enJSRV-HamJ2 UK | 0.8961039 |
| PP707044 JSRV-FR1167 FR | 0.8506494 | MF175069 enJSRV-HamM UK | 0.9025974 |
| PP707043 JSRV-FR987 FR | 0.8571429 | MF175070 enJSRV-KarJ UK | 0.9025974 |
| PP646154 JSRV-FR1481 FR | 0.8376623 | MF175071 enJSRV-KarM UK | 0.8961039 |
| OR729406 JSRV-SVUJY IN | 0.8571429 | EF680309 enJSRV-3 UK | 0.7922078 |
| KP691837 JSRV-C1 CH | 0.9675325 | EF680316 enJSRV-9 UK | 0.9025974 |
| M80216 JSRV-SA USA | 0.9025974 | EF680317 enJSRV-4 UK | N/A |
| PP707054 JSRV-FR2333 FR | 0.8896104 | EF680312 enJSRV-21 UK | 0.8311688 |
| PP707055 JSRV-FR1751 FR | 0.8896104 | EF680305 enJSRV-5 UK | N/A |
| MN161849 JSRV-DL37 IN | N/A | EF680307 enJSRV-10 UK | N/A |

N/A, not applicable.

**Table.S8.** Compare the percentage of amino acid similarity between NMJS12 and the reference strain Env.

| exJSRV | Similarity | enJSRV | Similarity |
| --- | --- | --- | --- |
| PP707042 JSRV-FR1468 FR | 0.995122 | AF136224 enJSRV5f16 SA | 0.9230769 |
| PP707041 JSRV-FR1037 FR | 0.9934959 | AF153615 enJSRV5.6A1 SA | 0.9247136 |
| PP707047 JSRV-FR989 FR | 0.9934959 | DQ838493 enJSRV-NM CH | 0.9279869 |
| PP707040 JSRV-FR985 FR | 0.9934959 | EF680296 enJSRV-13 UK | 0.9198036 |
| PP707038 JSRV-FR1466 FR | 0.995122 | EF680297 enJSRV-26 UK | 0.9247136 |
| PP707039 JSRV-FR1000 FR | 0.9934959 | EF680298 enJSRV-7 UK | 0.9263502 |
| PP707037 JSRV-FR1296 FR | 0.9918699 | EF680299 enSRV-15 UK | 0.9279869 |
| PP646155 JSRV-FR2369 FR | 0.9756098 | EF680300 enJSRV-16 UK | 0.9214402 |
| AF105220 JSRV-JSRV21 USA | 0.996748 | EF680301 enJSRV-18 UK | 0.9214402 |
| AF357971 JSRV-JS7 USA | 0.996748 | EF680302 enSRV-20 UK | 0.9263502 |
| MW883893 JSRV-Tonk1 IN | N/A | EF680303 enJSRV-11 UK | 0.9230769 |
| PP707053 JSRV-FR2334 FR | 0.9756098 | EF680306 enJSRV-8 UK | N/A |
| PP707052 JSRV-FR2529 FR | 0.9756098 | EF680308 enJSRV-14 UK | N/A |
| PP707051 JSRV-FR2332 FR | 0.9756098 | EF680310 enJSRV-2 UK | N/A |
| PP707050 JSRV-FR2054 FR | 0.9756098 | EF680311 enJSRV-1 UK | N/A |
| PP707049 JSRV-FR2586 FR | 0.9756098 | EF680313 enJSRV-23 UK | 0.919803 |
| PP707048 JSRV-FR2780 FR | 0.9756098 | MF175067 enJSRV-HamJ1 UK | 0.9296235 |
| PP707046 JSRV-FR1298 FR | 0.996748 | EF680314 enJSRV-25 UK | N/A |
| PP707045 JSRV-FR1762 FR | 0.996748 | MF175068 enJSRV-HamJ2 UK | 0.9296235 |
| PP707044 JSRV-FR1167 FR | 0.996748 | MF175069 enJSRV-HamM UK | 0.9296235 |
| PP707043 JSRV-FR987 FR | 0.995122 | MF175070 enJSRV-KarJ UK | 0.9296235 |
| PP646154 JSRV-FR1481 FR | 0.9918699 | MF175071 enJSRV-KarM UK | 0.9279869 |
| OR729406 JSRV-SVUJY IN | 0.996748 | EF680309 enJSRV-3 UK | N/A |
| KP691837 JSRV-C1 CH | 0.996748 | EF680316 enJSRV-9 UK | 0.9198036 |
| M80216 JSRV-SA USA | 0.9739838 | EF680317 enJSRV-4 UK | 0.9263502 |
| PP707054 JSRV-FR2333 FR | 0.9756098 | EF680312 enJSRV-21 UK | N/A |
| PP707055 JSRV-FR1751 FR | 0.9772358 | EF680305 enJSRV-5 UK | N/A |
| MN161849 JSRV-DL37 IN | N/A | EF680307 enJSRV-10 UK | N/A |

N/A, not applicable.
